# A transverse picoNewton force revealed in anisotropic Womersley flow

**DOI:** 10.1038/s41598-026-47474-x

**Published:** 2026-04-16

**Authors:** Khalid M. Saqr

**Affiliations:** Mechanical Engineering Department, College of Engineering and Technology, Arab Academy for Science, Technology, and Maritime Transport, Alexandria, 1029 Egypt

**Keywords:** Womersley flow, Endothelial cells, Blood anisotropy, PicoNewton forces, Engineering, Mathematics and computing, Physics

## Abstract

The fluid-dynamic quantity that regulates endothelial mechanotransduction remains unsettled. Wall Shear Stress (WSS) characterizes boundary traction but does not represent the volumetric inertial structure of arterial blood flow. Meanwhile, blood exhibits direction-dependent stress under physiological shear, suggesting that the classical Womersley solution of the Navier-Stokes equation may omit constitutive mechanisms relevant to near-wall dynamics. Here, I derive an anisotropic extension of Womersley flow by introducing a tensorial viscosity into the incompressible Navier–Stokes equations. By evaluating the nonlinear interaction of velocity and vorticity within a near-wall control volume, I demonstrate that anisotropic viscosity produces a non-trivial spectral signature in the transverse forcing, maintaining power across higher-order harmonics. While macroscopic geometric drivers dominate the bulk flow at the fundamental cardiac frequency, they are subject to significant inertial damping as the harmonic frequency increases. In contrast, the anisotropy-induced Lamb vector, sustained by the sharp gradients of the oscillatory boundary layer, evades this macroscopic attenuation. These findings define a geometry-independent baseline for multidirectional pulsatile dynamics and provide a theoretical basis for future spectral investigations of endothelial mechanobiology under high-frequency, near-wall inertial stimuli.

## Introduction

Endothelial cells (ECs) integrate mechanical cues from blood flow to regulate vascular tone, permeability, inflammation, and gene expression^1,2^. Despite decades of research, the identity of the fluid-dynamic quantity that initiates mechanotransduction remains unsettled. Wall shear stress (WSS) has long been used as the canonical descriptor of arterial hemodynamics, yet its predictive power is inconsistent across vascular beds and disease states. Atherosclerotic lesions preferentially form at *disturbed flow* regions where no universal WSS threshold exists^3,4^, and intracranial aneurysms remodel and rupture over wide ranges of shear values^5,6^. These discrepancies motivate a re-examination of the dynamical quantities that arise intrinsically from the Navier–Stokes equations.

Classical WSS theory posits that endothelial mechanotransduction is driven primarily by a scalar steady or time-averaged boundary traction; however, decades of clinical and computational studies show that WSS correlates poorly and inconsistently with focal atherosclerosis, aneurysm initiation, or rupture across vascular beds, modelling frameworks, and parameter choices^3–8^. These discrepancies persist even in large-sample CFD meta-analyses, which concluded that WSS fails to yield reproducible disease predictors and cannot robustly capture the spatiotemporal complexity of arterial flow^9,10^. In vivo measurements and high-fidelity simulations further demonstrate that physiological hemodynamics are neither steady nor quasi-laminar: geometric branching, curvature, and pulsatility generate broadband, non-Kolmogorov spectra, intermittent vortical structures, and coherent near-wall dynamics^11–13^. As a result, WSS-based descriptors collapse a multi-dimensional, vector-valued, spectrally rich environment into a single scalar or a few ad hoc derivatives (TAWSS, OSI, RRT), discarding phase information, velocity–vorticity interaction, and intermittency–features central to endothelial sensing.

The Gromeka–Lamb decomposition of the Navier–Stokes equation, $(\mathbf{u}\!\cdot \!\nabla )\mathbf{u} = \nabla (|\mathbf{u}|^{2}/2)-\mathbf{u}\times \boldsymbol{\omega }$, identifies the Lamb vector $\mathbf{u}\times \boldsymbol{\omega }$ as the non-conservative inertial field that quantifies local velocity–vorticity coupling. Experimental and theoretical evidence increasingly implicates these interactions, rather than boundary shear alone, in regulating endothelial mechanotransduction^8,11,14^. These observations motivate replacing WSS-based metrics with volumetric Lamb-vector descriptors that retain directionality, spectral content, and intermittency of near-wall forcing.

A second, independent source of complexity arises from blood rheology. Blood is a dense suspension, with red blood cells (RBCs) comprising 40–50% of its volume^15,16^. Under arterial shear rates, RBCs deform and align with the flow, producing anisotropic stress components^17^. RBC aggregation and near-wall platelet enrichment further amplify the direction-dependent rheological response^18^. Direct numerical simulations and rheometric measurements consistently demonstrate that the bulk stress tensor acquires off-diagonal components that cannot be represented by a scalar viscosity^19^. These findings motivate extending canonical pulsatile-flow theory to a tensorial constitutive law capable of supporting anisotropic stress–strain coupling.

In this study, the first anisotropic extension of Womersley’s solution is formulated. The extended solution demonstrates that introducing a viscosity tensor inevitably generates azimuthal momentum, bidirectional vorticity, and a transverse inertial force arising from the Lamb vector. Because the Womersley geometry is analytically tractable and free of geometric artefacts, it isolates anisotropy as the sole mechanism capable of generating swirl and transverse inertia in the exact Womersley configuration. This provides a controlled theoretical setting for examining how unsteady hemodynamics and anisotropic rheology can generate transverse inertial forcing relevant to endothelial-scale mechanics.

### Theoretical analysis

Wall shear stress (WSS) has long served as the primary causal proxy linking blood-flow dynamics to endothelial mechanobiology. However, WSS is not an intrinsic dynamical quantity of the Navier–Stokes equations. As established by tensor analysis in continuum mechanics^20^ and emphasized by Cherubini et al.^21^ and Saqr^9^, WSS is the projection of the deviatoric stress tensor onto the vessel wall. It is therefore a boundary traction, defined by a specific wall orientation, rather than a volumetric force arising directly from the governing equations. By construction, it contains no information about vorticity topology, rotational inertia, multidirectional flow organization, or harmonic structure of the bulk motion.

In controlled *in vitro* channel and microfluidic chambers^7,22^, endothelial phenotype is known to vary under conditions where the mean WSS is nominally identical but the bulk flow differs in recirculation, shear gradients, secondary motion, or waveform complexity. Endothelial cells do not consistently align with the mean WSS vector, and spatial shear gradients independently modulate signaling pathways^23^, demonstrating that $\tau _w$ alone does not uniquely determine endothelial response^24,25^. In theoretical terms, holding $\tau _w$ fixed does not necessarily fix the endothelial phenotype. This exposes a structural gap in the WSS paradigm: a boundary observable is being used as a causal bridge between two volumetric systems–blood-flow dynamics and endothelial mechanobiology.

The Navier–Stokes equations themselves identify a volumetric candidate for such a bridge. The nonlinear convective acceleration admits the Gromeka–Lamb decomposition^26,27^,

1 $$\begin{aligned} \mathbf{u}\cdot \nabla \mathbf{u} = \nabla \!\left( \tfrac{1}{2}|\mathbf{u}|^2\right) - \mathbf{u}\times \boldsymbol{\omega }, \end{aligned}$$

revealing the Lamb vector,

2 $$\begin{aligned} \boldsymbol{\ell } = \mathbf{u}\times \boldsymbol{\omega }, \qquad \boldsymbol{\omega }=\nabla \times \mathbf{u}, \end{aligned}$$

as the rotational inertial field associated with velocity–vorticity coupling. Unlike WSS, $\boldsymbol{\ell }$ is a three-dimensional volumetric quantity defined throughout the fluid domain. Two flows may share identical instantaneous $\tau _w$ while differing in vorticity structure or transverse kinematics; in such cases $\tau _w$ coincides, but $\boldsymbol{\ell }$ differs in magnitude, phase, and harmonic composition. The Lamb vector therefore encodes inertia-driven flow organization that is invisible to viscous traction alone.

In the general three-dimensional cylindrical case, $\boldsymbol{\ell }=(\ell _r,\ell _\theta ,\ell _z)$ depends on the full velocity field $\mathbf{u}=(u_r,u_\theta ,u_z)$ and vorticity $\boldsymbol{\omega }=(\omega _r,\omega _\theta ,\omega _z)$, with components

3 $$\begin{aligned} \ell _r = u_\theta \omega _z - u_z \omega _\theta , \quad \ell _\theta = u_z \omega _r - u_r \omega _z, \quad \ell _z = u_r \omega _\theta - u_\theta \omega _r . \end{aligned}$$

The present work considers the analytically tractable Womersley limit: a straight, rigid, axisymmetric tube with fully developed axial flow. Under these constraints ($u_r=0$, $\partial _\theta =0$), the Lamb vector reduces to a single radial component,

$$\ell _r = u_\theta \,\omega _z - u_z\,\omega _\theta ,$$

with $\omega _\theta =-\partial _r u_z$ and $\omega _z=\frac{1}{r}\partial _r(r u_\theta )$, yielding

4 $$\begin{aligned} \ell _r = u_\theta \,\frac{1}{r}\frac{\partial }{\partial r}(r u_\theta ) + u_z\,\frac{\partial u_z}{\partial r}. \end{aligned}$$

The associated transverse inertial force per unit volume is $f_r=\rho \,\ell _r$.

In the classical isotropic Womersley solution, $u_\theta \equiv 0$, and the transverse inertial contribution collapses to the centripetal term $u_z\,\partial _r u_z$. No swirl, no axial vorticity, and no bidirectional transverse mode are permitted. The aim of the present study is to determine whether admitting viscosity anisotropy–supported by experimental and computational evidence in RBC suspensions^28–31^—is sufficient, within this theoretical limit of arterial hemodynamics, to activate a non-zero transverse Lamb-vector mode in the absence of geometric secondary flows. By extending the Womersley solution to a tensorial constitutive law, the analysis isolates the minimal mechanism through which anisotropic shear coupling can generate azimuthal momentum, bidirectional vorticity, and a finite near-wall transverse inertial force. In doing so, it introduces a volumetric, equation-derived descriptor that complements WSS and emerges directly from the governing dynamics of pulsatile flow.

## Materials and methods

In the analysis used to demonstrate the inertial origin of the picoNewton forces, the pulsatile flow of blood is treated as that of an incompressible fluid flowing through a rigid, straight cylindrical vessel of radius *R*. The flow is driven by a time-varying axial pressure gradient and is assumed to be axisymmetric (i.e., derivatives with respect to the azimuthal angle $\theta$ are zero, $\partial /\partial \theta = 0$) with no radial velocity component ($u_r=0$). These assumptions reduce the problem to two non-zero velocity components: an axial component, $u_z$, and an azimuthal (swirl) component, $u_\theta$. In a straight cylindrical vessel with a fully developed axial pressure gradient, the incompressible continuity equation,

$$\frac{1}{r}\frac{\partial }{\partial r}(r u_r) + \frac{\partial u_z}{\partial z} = 0,$$

reduces to $\partial u_z/\partial z = 0$ under the standard Womersley assumption of axial invariance. This enforces $u_r = 0$ throughout the domain: any non-zero radial velocity would violate continuity in a straight tube unless accompanied by axial development. Thus, $u_r=0$ is a defining property of the classical Womersley limit, which represents the canonical geometry for isolating pulsatile shear transport in large arteries. This minimal-model setting intentionally removes geometric sources of secondary flow (curvature, taper, branching), ensuring that the emergence of swirl and transverse inertia in the present formulation arises solely from the anisotropic constitutive coupling rather than from vessel geometry.

### Model assumptions and scope

The formulation developed in this work is based on the classical straight-tube Womersley geometry, in which the vessel wall is rigid and the flow is axisymmetric and fully developed. These assumptions imply $u_r = 0$ and $\partial _\theta (\cdot )=0$, so that transport in the radial and azimuthal directions arises exclusively from shear coupling rather than from geometry-induced secondary motion. In a straight cylindrical vessel this constraint is a direct consequence of the continuity equation: with no variation in $\theta$ or *z*, the divergence-free condition forces $u_r$ identically to zero. The resulting kinematic structure isolates the component of inertial dynamics that is intrinsic to the constitutive law rather than to curvature or tortuosity.

This reduced setting serves as a minimal analytical model in which viscosity anisotropy is the sole mechanism capable of generating azimuthal momentum, axial–azimuthal vorticity in an axisymmetric straight cylinder. Secondary flows associated with curvature, branching, or torsion—including the familiar Dean-type vortical patterns—do not occur under these constraints and would introduce additional sources of transverse motion unrelated to the constitutive anisotropy. By excluding such geometric effects, the present model isolates and characterizes the fundamental inertial mode that emerges directly from the tensorial stress–strain coupling. The full three-dimensional expressions for the Lamb vector and vorticity fields are given in Supplementary Information Note SI1, with the reduced Womersley form obtained as a special case under these symmetry and regularity conditions.

### Governing equations

Under the stated assumptions, the incompressible Navier–Stokes equations in cylindrical coordinates $(r, \theta , z)$ simplify to the following momentum equations for the axial and azimuthal directions:

5 $$\begin{aligned} \rho \frac{\partial u_z}{\partial t}&= -\frac{\partial p}{\partial z} + \frac{1}{r}\frac{\partial }{\partial r}(r\tau _{zr}), \end{aligned}$$

6 $$\begin{aligned} \rho \frac{\partial u_\theta }{\partial t}&= \frac{1}{r^2}\frac{\partial }{\partial r}(r^2\tau _{\theta r}), \end{aligned}$$

where $\rho$ is the fluid density, *p* is the pressure, *t* is time, and $\tau _{zr}$ and $\tau _{\theta r}$ are the shear stress components, which are determined by the fluid’s constitutive law.

### Anisotropic constitutive model

Blood is modeled as an anisotropic Newtonian fluid. This implies a linear relationship between stress and strain rate, but with a viscosity that depends on direction. The relationship is encapsulated by a fourth-rank viscosity tensor, which for this flow configuration simplifies to a $2 \times 2$ matrix relating the relevant shear stresses to the strain rates:

7 $$\begin{aligned} \begin{bmatrix} \tau _{zr}\\ \tau _{\theta r} \end{bmatrix} = \rho \begin{bmatrix} \nu _{zz} & \nu _{z\theta }\\ \nu _{\theta z} & \nu _{\theta \theta } \end{bmatrix} \begin{bmatrix} \partial _r u_z \\ \left( \partial _r u_\theta - \dfrac{u_\theta }{r}\right) \end{bmatrix}. \end{aligned}$$

Here, the diagonal terms $\nu _{zz}$ and $\nu _{\theta \theta }$ represent the kinematic viscosities for axial and azimuthal shear, respectively. The off-diagonal terms, $\nu _{z\theta }$ and $\nu _{\theta z}$, are the crucial anisotropy coefficients that couple axial shear to azimuthal stress and vice versa. For an isotropic fluid, these off-diagonal terms are zero and $\nu _{zz}=\nu _{\theta \theta }$, recovering the standard Newtonian model. The viscosity matrix is assumed to be positive-definite to ensure dissipative mechanics. A complete derivation of the anisotropic momentum equations in cylindrical coordinates, including the reduction of the fourth–rank viscosity tensor to the $2\times 2$ form used here, is provided in Supplementary Information note SI1.

It is important to clarify that the anisotropic Newtonian tensor adopted here represents the macroscopic, continuum-scale limit in large arteries, and is not intended to reproduce the thixotropic elasto–visco–plastic (TEVP) behavior observed in microcirculatory flows^32^. The TEVP framework is formulated for steady blood flow in microtubes of radius $R \approx 10$–$80~\upmu \textrm{m}$, where the cell-free layer occupies a substantial fraction of the lumen and migration-, aggregation-, and yield-stress effects dominate the constitutive response. These microscale mechanisms are negligible in the arterial (macrotube) limit considered here: the CFL thickness is *O*(3–$5~\upmu \textrm{m})$ ($<0.1\%$ of the vessel radius), the hematocrit is effectively uniform across the lumen, and arterial shear rates suppress rouleaux formation. The anisotropic tensor used here should thus be interpreted as an effective continuum representation of near-wall stress anisotropy in large vessels, rather than a microscale TEVP model appropriate for capillary or microtube rheology.

### Frequency-domain formulation

The driving pressure gradient is periodic and can be decomposed into a Fourier series:

$$-\frac{\partial p}{\partial z}(t) = \Re \left[ \sum _{h=0}^{H} \hat{G}_h e^{i\omega _h t} \right] ,$$

where $\hat{G}_h$ are the complex amplitudes of the pressure gradient harmonics at angular frequencies $\omega _h = h\omega _0$. Here, *h* is the integer harmonic index, and $\omega _0$ is the fundamental angular frequency corresponding to the heart rate. Due to the linearity of the governing equations, the velocity components can be similarly decomposed: $u_j(r,t) = \Re [\sum _h \hat{u}_{j,h}(r) e^{i\omega _h t}]$, where $\hat{u}_{j,h}$ are the complex velocity amplitudes for each component $j \in \{z, \theta \}$. To construct the frequency–domain system, the axial pressure gradient is represented as a truncated Fourier series,

$$\hat{G}(t) = \sum _{h=0}^{H} \hat{G}_h \, e^{\, i h \,\omega _0 t},$$

where $\hat{G}_h \in \mathbb {C}$ are the complex amplitudes of the waveform and $H$ is the maximum harmonic retained. In all computations presented in this study, $H$ was selected such that the root–mean–square reconstruction error between the measured waveform and its truncated Fourier approximation remained below $10^{-3}$. This choice ensures that each physiological waveform is resolved with spectral fidelity. For each harmonic $h$, an independent $2(N+1)\times 2(N+1)$ block system is assembled and solved. After solving for the complex velocity amplitudes $\hat{U}_{z,h}^*(r)$ and $\hat{U}_{\theta ,h}^*(r)$, the time–domain fields are reconstructed by Fourier synthesis:

$$u_j(r,t) = \Re \left( \sum _{h=0}^{H} \hat{U}_{j,h}^*(r) \, e^{\, i h \,\omega _0 t} \right) , \qquad j\in \{z,\theta \},$$

which exactly matches the numerical implementation in the open-source solver. Substituting the constitutive law into the momentum equations and transforming to the frequency domain yields a system of coupled ordinary differential equations for the complex velocity amplitudes of each harmonic (subscript *h* and frequency $\omega = \omega _h$ are implied for clarity):

8 $$\begin{aligned} i\omega \hat{u}_z&= \frac{\hat{G}}{\rho } + \nu _{zz} L_0 \hat{u}_z + \nu _{z\theta } L_1 \hat{u}_\theta , \end{aligned}$$

9 $$\begin{aligned} i\omega \hat{u}_\theta&= \nu _{\theta z} L_0 \hat{u}_z + \nu _{\theta \theta } L_1 \hat{u}_\theta , \end{aligned}$$

where $i = \sqrt{-1}$. The linear differential operators $L_0$ and $L_1$ are defined as:

10 $$\begin{aligned} L_0 f(r)&:= \frac{1}{r}\frac{d}{dr}\left( r \frac{df}{dr}\right) = \frac{d^2f}{dr^2} + \frac{1}{r}\frac{df}{dr}, \end{aligned}$$

11 $$\begin{aligned} L_1 f(r)&:= \frac{1}{r^2}\frac{d}{dr}\left( r^2 \left( \frac{df}{dr} - \frac{f}{r}\right) \right) = \frac{d^2f}{dr^2} + \frac{1}{r}\frac{df}{dr} - \frac{f}{r^2}. \end{aligned}$$

The correspondence between these analytical operators and their spectral discretizations is demonstrated in Supplementary Information note SI3, which includes the explicit construction of the differentiation matrices. The assembly of the harmonic-by-harmonic sparse linear systems and their solution are described in Supplementary Section SI3.

### Dimensionless formulation

The system is non-dimensionalized using the vessel radius *R*, a characteristic velocity scale $U_0 = |\hat{G}_0|R^2/(\rho \nu _{zz})$, and the fundamental frequency $\omega _0$. The dimensionless variables (marked with an asterisk) are $r^*=r/R$, $\hat{U}^*=\hat{u}/U_0$, and $t^*=\omega _0 t$. This introduces the Womersley number, $\alpha = R\sqrt{\omega _0/\nu _{zz}}$, which represents the ratio of transient inertial forces to viscous forces.

For each harmonic *h*, the governing equations in dimensionless form become:

12 $$\begin{aligned} i f_h \alpha ^2 \hat{U}_z^*&= a_h + L_0^* \hat{U}_z^* + \beta L_1^* \hat{U}_\theta ^*, \end{aligned}$$

13 $$\begin{aligned} i f_h \alpha ^2 \hat{U}_\theta ^*&= \gamma L_0^* \hat{U}_z^* + \delta L_1^* \hat{U}_\theta ^*, \end{aligned}$$

where $f_h = \omega _h/\omega _0 = h$ is the dimensionless frequency and $a_h = \hat{G}_h R^2/(\rho \nu _{zz}U_0)$ is the dimensionless pressure gradient amplitude. The system is governed by three dimensionless anisotropy ratios:

$$\beta = \frac{\nu _{z\theta }}{\nu _{zz}}, \quad \gamma = \frac{\nu _{\theta z}}{\nu _{zz}}, \quad \delta = \frac{\nu _{\theta \theta }}{\nu _{zz}}.$$

### Solution method

The coupled system of dimensionless equations is solved for each harmonic using a spectral collocation method encapsulated in a Python class, WomersleySolver.^1^ The radial domain $r^*\in [0,1]$ is discretized using *N* Chebyshev–Gauss–Lobatto nodes, which allows for spectrally accurate differentiation matrices approximating the operators $L_0^*$ and $L_1^*$. In essence, this method, as explained in Supplementary Information note SI3, approximates the unknown velocity profiles as a high-order, smooth mathematical function (a polynomial), which allows for exceptionally accurate calculation of velocity gradients and stresses.

For each harmonic, the discretized governing equations are formulated as a $2(N+1) \times 2(N+1)$ sparse block-matrix system:

$$\begin{bmatrix} \mathbf{A}_{zz} & \mathbf{A}_{z\theta } \\ \mathbf{A}_{\theta z} & \mathbf{A}_{\theta \theta } \end{bmatrix} \begin{bmatrix} \hat{\mathbf{U}}_z^* \\ \hat{\mathbf{U}}_\theta ^* \end{bmatrix} = \begin{bmatrix} \mathbf{b}_z \\ \mathbf{0} \end{bmatrix},$$

where $\hat{\mathbf{U}}^*$ are vectors of the complex velocity amplitudes at the collocation points, and the block matrices $\mathbf{A}_{ij}$ contain the discretized differential operators and inertial terms.

The system is closed with physically-motivated boundary conditions, which are imposed directly into the matrix system via row replacement. The no-slip conditions are enforced at the vessel wall ($r^*=1$). At the centerline ($r^*=0$), symmetry and regularity are enforced: $\frac{d\hat{U}_z^*}{dr^*}(0)=0$ and $\hat{U}_\theta ^*(0)=0$. The resulting sparse, complex-valued linear system is solved for each harmonic using direct LU factorization via scipy.sparse.linalg.spsolve. The enforcement of the regularity and no-slip boundary conditions by row-replacement within the spectral differentiation matrices is shown in Supplementary Section SI3, along with the exact matrix structures used in the solver.

### Definition and interpretation of the endothelial transverse force

Using the Gromeka–Lamb decomposition^26,27^, the nonlinear convective acceleration can be written as

$$\mathbf{u}\cdot \nabla \mathbf{u} = \nabla \!\left( \tfrac{1}{2}|\mathbf{u}|^2\right) - \boldsymbol{\ell }, \qquad \boldsymbol{\ell }=\mathbf{u}\times \boldsymbol{\omega }, \quad \boldsymbol{\omega }=\nabla \times \mathbf{u}.$$

Here $\boldsymbol{\ell }$ is a volumetric inertial *acceleration* field. The corresponding inertial force density is

$$\mathbf{f}(r,t)=\rho \,\boldsymbol{\ell }(r,t)\qquad [\mathrm {N/m^3}].$$

Under axisymmetry ($u_r=0$, $\partial _\theta =0$), the Lamb vector reduces to a single radial component

14 $$\begin{aligned} \ell _r = u_\theta \,\frac{1}{r}\frac{\partial }{\partial r}(r u_\theta ) + u_z\,\frac{\partial u_z}{\partial r}, \end{aligned}$$

and the transverse inertial force density is $f_r=\rho \,\ell _r$. In an isotropic fluid, $u_\theta =0$ identically, so the transverse inertial contribution collapses to $\ell _r^{\textrm{iso}}=\rho u_z\,\partial _r u_z$. Viscosity anisotropy activates the full structure by generating $u_\theta \ne 0$ and $\omega _z\ne 0$, yielding a transverse mode forbidden in the classical solution.

### Locality and cumulative endothelial forcing

Endothelial cells do not exist as fluid particles; rather, they form a boundary layer that experiences the cumulative effect of the momentum flux in the fluid column directly above them. To accurately capture this transverse load, the local near-wall inertial forcing estimate, $F_{\textrm{EC}}(t)$, is evaluated using a control-volume approach. We define an endothelial-scale fluid “pillbox” of thickness $\delta _{\textrm{EC}} = V_{\textrm{EC}} / A_{\textrm{EC}}$ adjacent to the wall ($r=R$), where $A_{\textrm{EC}}$ is the characteristic cell footprint and $V_{\textrm{EC}}$ is the cell volume. Here, $\delta _{\textrm{EC}}$ denotes the thickness of the near-wall endothelial control volume and should not be confused with the harmonic index *h* used in the frequency-domain formulation.

Crucially, because the Lamb vector $\boldsymbol{\ell } = \mathbf{u} \times \boldsymbol{\omega }$ is a non-linear product of the velocity and vorticity fields, the frequency-domain solutions for $\hat{\mathbf{u}}$ and $\hat{\boldsymbol{\omega }}$ cannot simply be multiplied in the complex plane. Instead, the physical (real-valued) velocity and vorticity fields are first reconstructed in the time domain via Fourier synthesis to capture all cross-harmonic interactions. The physical transverse force density, $f_r(r,t) = \rho (\mathbf{u} \times \boldsymbol{\omega })_r$, is then calculated. The quantity reported in the manuscript is the localized near-wall inertial proxy obtained by integrating this field over a thin endothelial-scale pillbox control volume, as derived in Supplementary Information Note SI2:

15 $$\begin{aligned} F_{\textrm{EC}}(t) = A_{\textrm{EC}} \int _{R-\delta _{\textrm{EC}}}^{R} |f_r(r,t)| \, dr \end{aligned}$$

The exact wall-force balance is given in Supplementary Information Note SI2, Eqs. (SI32) and (SI35), while the reported proxy is defined in Eqs. (SI38)–(SI40). Statistical distinctness of the resulting force distributions was verified using a non-parametric Kruskal–Wallis H-test. Furthermore, a Fast Fourier Transform (FFT) was applied to the integrated signal $F_{\textrm{EC}}(t)$ to quantify the spectral redistribution caused by the nonlinear inertial coupling.

#### Competitive spectral scaling and inertial filtering of transverse forces

While this study primarily addresses the analytical extension of the exact Womersley solution in the straight-tube limit, the mechanobiological relevance of the resulting force field must be contextualized against the transverse drivers prevalent in complex arterial geometries. In curved vessel segments, curvature introduces a geometry-driven transverse inertial loading that is absent in the straight-tube limit. For the present comparison, this geometric contribution is represented only as a conservative order-of-magnitude upper bound, written as the sum of a curvature-induced centrifugal scale associated with the bulk axial motion and a cross-stream correction associated with curvature-driven secondary motion:

16 $$\begin{aligned} f_{r,c}(r,t) = \rho \left( \frac{u_z(r,t)^2}{\mathcal {R}} + \frac{u_s(r,t)^2}{r} \right) \end{aligned}$$

where $\mathcal {R}$ is the local radius of curvature and $u_s$ denotes the characteristic secondary-flow velocity scale. In the comparisons reported below, the geometric forcing is evaluated conservatively using the macroscopic bulk-velocity scale together with the near-wall estimate $r\approx R$, which gives

17 $$\begin{aligned} f_{r,c}(r,t) \lesssim \rho \, u_z(r,t)^2 \,\frac{1+\kappa }{R}, \qquad \kappa =\frac{R}{\mathcal {R}}. \end{aligned}$$

This estimate is used only to bound the potential magnitude of curvature-driven transverse loading before the inertial frequency filtering discussed below is applied. $\mathcal {R}$. In the limit of high curvature ($\kappa = R/\mathcal {R} \sim 0.3-0.7$), the transverse pressure gradient $\partial _r p \approx \rho u_z^2 / \mathcal {R}$ is balanced by the convective inertia of the secondary flow, $\rho (u_s \cdot \nabla ) u_s \sim \rho u_s^2/R$. This inertial scaling dictates that $u_s \sim u_z \sqrt{\kappa }$, implying that in segments such as the aortic arch or internal carotid artery, the secondary flow magnitude approaches that of the primary axial flow ($u_s \sim u_z$). The corresponding scaling form may be written as: 

18 $$\begin{aligned} f_{r,c}(r,t) \approx \rho \, u_z(r,t)^2 \left( \frac{\kappa }{R} + \frac{1}{r} \right) \end{aligned}$$

This bulk geometric driver is evaluated against the constitutive Gromeka-Lamb force density, $f_{r,a}(r,t) = \rho (\mathbf{u} \times \boldsymbol{\omega })_r$, localized within the Womersley boundary layer. To compare their cumulative effects on the vessel wall, both volumetric fields are integrated across a near-wall endothelial control volume $V_{\textrm{EC}}$ of thickness $\delta _{\textrm{EC}}$ to yield the integrated picoNewton-scale loads $F_{c,\textrm{EC}}$ and $F_{a,\textrm{EC}}$:

19 $$\begin{aligned} F_{c,\textrm{EC}}(t) = A_{\textrm{EC}} \int _{R-\delta _{\textrm{EC}}}^{R} |f_{r,c}(r,t)| \, dr, \qquad F_{a,\textrm{EC}}(t) = A_{\textrm{EC}} \int _{R-\delta _{\textrm{EC}}}^{R} |f_{r,a}(r,t)| \, dr \end{aligned}$$

The physical distinction between these mechanisms is revealed through their frequency scaling, which is now summarized explicitly in Supplementary Note SI1. For the anisotropic near-wall mechanism, the harmonic Womersley parameter satisfies $\alpha _h=\sqrt{f_h}\,\alpha$ and the associated oscillatory layer thickness scales as $\delta _{\textrm{W},h}=O(h^{-1/2})$; see supplementary Eqs. (SI19)–(SI22). For the geometric comparison, the bulk curvature-driven cross-stream response is treated only through the first-order inertial estimate $\hat{u}_{s,h}=O(\hat{f}_{r,c,h}/(\rho \omega _h))$, so that its transfer magnitude carries a 1/*h* attenuation factor; see supplementary Eqs. (SI23)–(SI24). Thus, the geometric benchmark is progressively suppressed at high harmonic number, whereas the anisotropic near-wall force is evaluated directly from the computed velocity–vorticity fields within the shrinking oscillatory layer. The sustained higher-harmonic content of the anisotropic force is therefore an observed outcome of the anisotropic Womersley solution rather than an imposed transfer function.

### Parameter ranges and physiological values

The dimensionless Womersley number, $\alpha = R\sqrt{\omega _0/\nu _{zz}}$, varies across the arterial tree. In this study, $\alpha$ was computed from the radii and heart rates associated with each waveform in the Willemet et al. database^33^. The six arterial segments analysed span approximately $\alpha \approx 3 \; \text {(brachial)} \quad \text {to} \quad \alpha \approx 22 \;\text {(aortic root)}$, thus covering viscous-, transitional-, and inertia-dominated flow regimes.

The anisotropic viscosity tensor is parameterized via

20 $$\begin{aligned} \beta = \nu _{z\theta }/\nu _{zz}, \quad \gamma = \nu _{\theta z}/\nu _{zz}, \quad \delta = \nu _{\theta \theta }/\nu _{zz}. \end{aligned}$$

Here and throughout, $\delta$ denotes the constitutive anisotropy ratio $\nu _{\theta \theta }/\nu _{zz}$. To avoid notational ambiguity, the oscillatory Womersley boundary-layer thickness is denoted separately by $\delta _{\textrm{W}}$. Although direct in vivo measurements of these components are unavailable, evidence from numerical simulations and microstructural rheology of deformable RBC suspensions indicates that off-diagonal (“cross”) stress components remain modest compared to shear stresses, on the order of a few to a few tens of percent under physiological conditions^31^. Therefore, the parameter space explored here uses $\beta , \gamma \in [-0.1, 0.1], \text {and } \delta \in [0.9, 1.1],$ bracketing plausible, physiologically relevant anisotropy. The isotropic limit is recovered at $\beta = \gamma = 0$, $\delta = 1$. To ensure reproducibility, Table 1 lists for each arterial segment the vessel radius *R*, the corresponding Womersley number $\alpha$, and the amplitudes of the first six harmonics of the physiological pressure waveform used in the simulations.

**Table 1:** Arterial segments and physiological parameters used in the multi-harmonic waveform analysis.

Artery/segment	Radius *R* (m)	$\alpha$	Waveform amplitudes (6 harmonics)
Aortic Root	0.015	22.03	[1.00, 0.82, 0.54, 0.33, 0.24, 0.17]
Thoracic Aorta	0.012	17.62	[1.00, 0.76, 0.45, 0.28, 0.20, 0.12]
Femoral	0.004	5.87	[1.00, 0.58, 0.10, -0.17, 0.05, 0.04]
Carotid	0.0035	5.14	[1.00, 0.63, 0.31, 0.15, 0.10, 0.06]
Iliac	0.0045	6.61	[1.00, 0.51, 0.12, -0.11, 0.05, 0.03]
Brachial	0.002	2.94	[1.00, 0.49, 0.16, -0.05, 0.02, 0.01]

R is the vessel radius, $\omega _0$ is the fundamental angular frequency of the cardiac cycle, and $\alpha = R \sqrt{\omega _0/\nu }$ is the corresponding Womersley number. Waveform amplitudes are taken directly from the digitized pressure gradients used in the simulation code. Source: Willemet et al.^33^.

### Grid-independence assessment

**Fig. 1:**
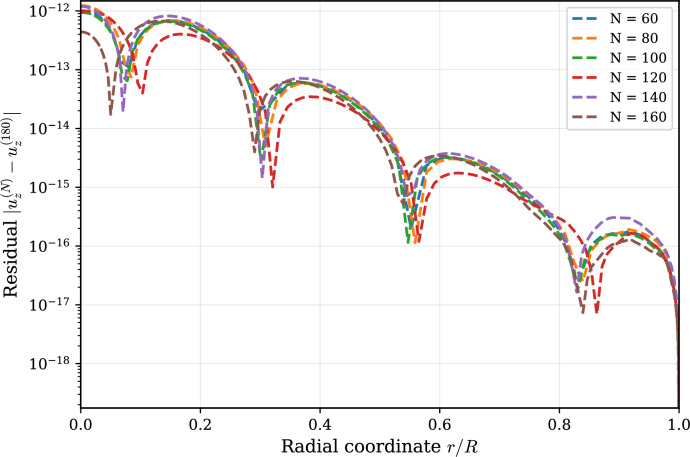
Grid-independence assessment using spectral-consistent pointwise residuals $|u_z^{(N)}(r)-u_z^{(180)}(r)|$ evaluated at peak phase $t=0.25$. Residual magnitudes decrease uniformly with refinement and collapse below $10^{-14}$ for $N \ge 140$, confirming grid independence of the resolution used in the study ($N=150$).

The spectral resolution was verified through successive grid refinement using Chebyshev–Gauss–Lobatto discretizations $N=\{60,80,100,120,140,160,180\}$. For a representative physiological case (thoracic aorta, $\beta =0.1$), the fully reconstructed axial velocity profile $u_z(r,t)$ was evaluated at peak phase $t=0.25$. The solution computed on the finest tested grid ($N=180$) was treated as reference and evaluated on coarser grids using barycentric interpolation to ensure spectral consistency. Figure 1 shows the pointwise residual $|u_z^{(N)}(r)-u_z^{(180)}(r)|$ across the radial domain, as further explained in Supplementary Information Note SI4. Residual magnitudes decrease uniformly with refinement and fall below $10^{-14}$ for $N\ge 140$, indicating that the production resolution ($N=150$) is safely within the grid-independent regime bracketed by $N=140$ and $N=160$.

### Validation against the classical Womersley solution

To verify the correctness of the numerical implementation, the solver was benchmarked against the classical isotropic Womersley solution, for which closed-form expressions for $u_z(r,t)$ are available. Setting the anisotropy coefficients to their isotropic values ($\beta =\gamma =0$, $\delta =1$) decouples the governing equations and reduces the model to a single linear ODE for each harmonic, whose analytical solution is

$$u_z(r,t) = \Re \!\left[ \frac{\hat{G}}{i\omega \rho } \left( 1 - \frac{J_0(\lambda r)}{J_0(\lambda )} \right) e^{i\omega t} \right] , \qquad \lambda = \sqrt{i}\,\alpha .$$

Using this expression as a reference, the $L^2$ and $L^\infty$ errors between the analytical axial velocity and the numerical solution were obtained from the spectral collocation method. For a representative range of Womersley numbers ($3 \le \alpha \le 20$), the error decreased spectrally with the number of collocation points. With $N=150$, the $L^\infty$ error was below $10^{-8}$ across all tested frequencies, confirming that the differentiation matrices, boundary-condition enforcement, and harmonic-by-harmonic assembly are implemented correctly. The numerical phase lag and amplitude of the oscillatory velocity profiles were visually indistinguishable from the analytical forms at this resolution.

A second validation was performed by verifying the recovery of the isotropic limit of the Lamb-vector forcing. When $\beta ,\gamma \rightarrow 0$, the solver returns $u_\theta \equiv 0$ and the transverse Lamb-vector component reduces to the expected centripetal term $u_z\,\partial _r u_z$. This behavior was reproduced numerically to machine precision for all harmonics, confirming that the anisotropic coupling enters the formulation solely through the intended off-diagonal viscosity terms and that no spurious swirl is generated by the discretization.

Together, these benchmarks demonstrate that the solver reproduces the exact isotropic Womersley solution, exhibits spectral convergence, and preserves the correct physical limit as the anisotropy parameters are varied. The anisotropic results presented in the following sections therefore represent the intended extension of the classical theory rather than numerical artefacts.

## Results

The numerical solution of the anisotropic Womersley flow equations reveals a complex interplay between fluid inertia, viscosity, and anisotropic effects. The results are presented in two stages: first, a parametric analysis of the system’s fundamental response to a single sinusoidal pressure gradient, and second, an analysis of the flow under realistic, multi-harmonic physiological waveforms. The consistency between the analytical expressions for $(u_\theta ,\omega _\theta ,\omega _z)$ and their numerical evaluation using Chebyshev spectral differentiation, as well as the construction of the Lamb vector at near-wall collocation points, is demonstrated in Supplementary Information note SI3.

### Sinusoidal flow analysis

**Fig. 2:**
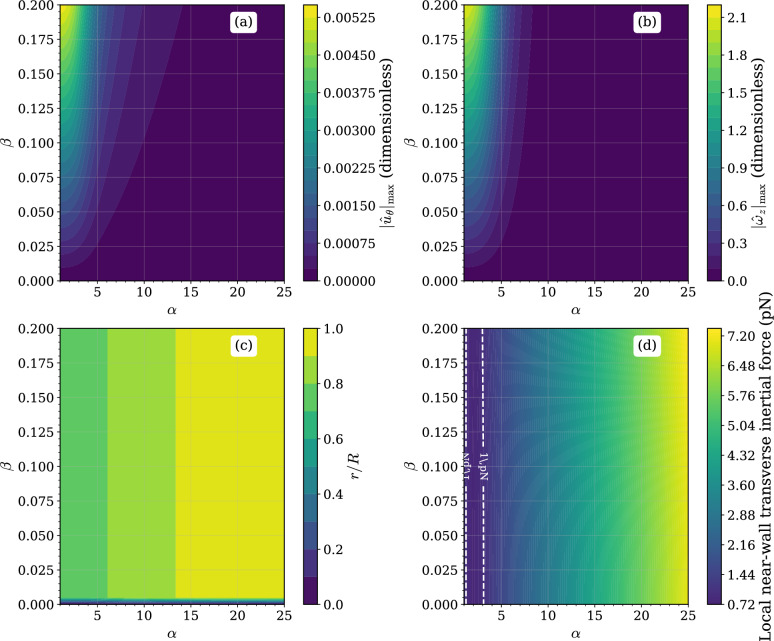
Parametric signatures of anisotropic Womersley flow under mono-harmonic forcing. (**a**) Peak azimuthal velocity magnitude $|\hat{u}_\theta |_{\max }$ as a function of the Womersley number $\alpha$ and anisotropy ratio $\beta$. (**b**) Corresponding peak axial vorticity magnitude $|\hat{\omega }_z|_{\max }$. (**c**) Radial location of the peak azimuthal velocity, expressed as a fraction of the vessel radius *r*/*R*. (**d**) Local near-wall transverse inertial force derived from the Lamb vector $u \times \omega$, evaluated at the collocation point nearest to the vessel wall and expressed in picoNewtons (pN). Logarithmic colour scaling is used in (**d**); dashed contours indicate reference force levels.

The system’s response to a mono-harmonic driving pressure was investigated across a wide physiological range of Womersley numbers ($\alpha$) and anisotropy ratios ($\beta$). The results, summarized in Fig. 2, detail a clear causal chain from the imposed anisotropy to the generation of a transverse force.

The magnitude of the anisotropy-induced swirl velocity, $|\hat{U}_\theta |_\text {max}$, is shown in Fig. 2a. The swirl velocity is zero for isotropic fluids ($\beta =0$) and its magnitude increases monotonically with the anisotropy ratio $\beta$. For a fixed $\beta$, the swirl magnitude exhibits a non-monotonic dependence on the Womersley number, reaching a maximum for intermediate values in the range of $5< \alpha < 12$. This secondary flow generates an axial component of vorticity, $|\hat{\omega }_z|_\text {max}$, whose magnitude is presented in Fig. 2b. Similar to the swirl velocity, the axial vorticity scales with $\beta$. However, its peak response is localized at a lower Womersley number, $\alpha \approx 4$. Figure 2c details the radial position of the peak swirl velocity. The location is shown to be a strong function of $\alpha$ and largely independent of $\beta$. At low $\alpha$, the peak is located near $r/R \approx 0.72$. As $\alpha$ increases, the peak progressively shifts towards the vessel wall, reaching $r/R \approx 0.96$ for $\alpha =25$. The resulting near-wall radial inertial forcing estimate is presented in Fig. 2d where its relation to the exact wall-traction balance is discussed in Supplementary Note SI2. The force magnitude increases with both $\beta$ and $\alpha$. Unlike the swirl velocity and vorticity, the force does not exhibit a peak at intermediate $\alpha$ but increases monotonically across the entire range investigated. For physiologically relevant anisotropy ($\beta > 0.05$), the force magnitude becomes comparable to picoNewton-scale force levels reported in *in vitro* mechanosensitivity studies for $\alpha > 5$ and can reach values greater than 6 pN for high $\alpha$ and $\beta$. This comparison is intended only as a magnitude reference.

**Fig. 3:**
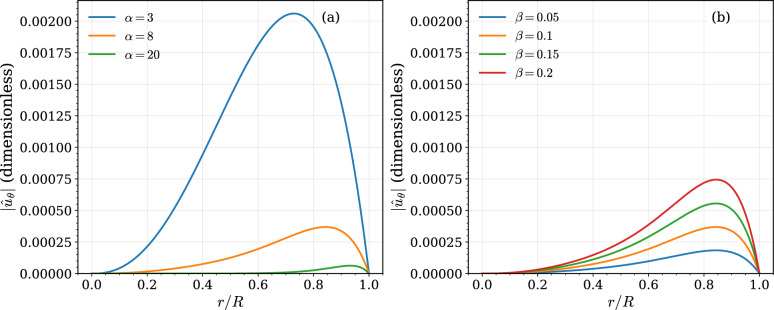
Radial profiles of the azimuthal velocity magnitude under mono-harmonic forcing. (**a**) Dependence on the Womersley number $\alpha$ at fixed anisotropy ratio $\beta =0.1$. (**b**) Dependence on the anisotropy ratio $\beta$ at fixed Womersley number $\alpha =8$. The azimuthal velocity $|\hat{u}_\theta |$ is shown in dimensionless form, and the radial coordinate is normalized by the vessel radius *R*.

A more detailed view of the swirl velocity profiles is provided in Fig. 3. Figure 3a shows the effect of the Womersley number at a fixed anisotropy of $\beta =0.1$. At low frequency ($\alpha =3$), the velocity profile is smooth and parabolic-like. At $\alpha =8$, near the peak of the system’s response, the profile is fuller and has the largest magnitude. At high frequency ($\alpha =20$), the profile becomes blunt in the core with a thin, steep boundary layer near the wall, consistent with classic Womersley theory. Figure 3b shows the effect of the anisotropy ratio at a fixed $\alpha =8$. The results confirm that the magnitude of the swirl velocity profile scales linearly with $\beta$ while the characteristic shape of the profile is preserved.

### Physiological waveform analysis

To investigate the relevance of the anisotropic force under realistic conditions reported by Willemet et al.^33^, the analysis was extended to six representative human arteries, each defined by its unique physiological pressure waveform and Womersley number.

**Fig. 4:**
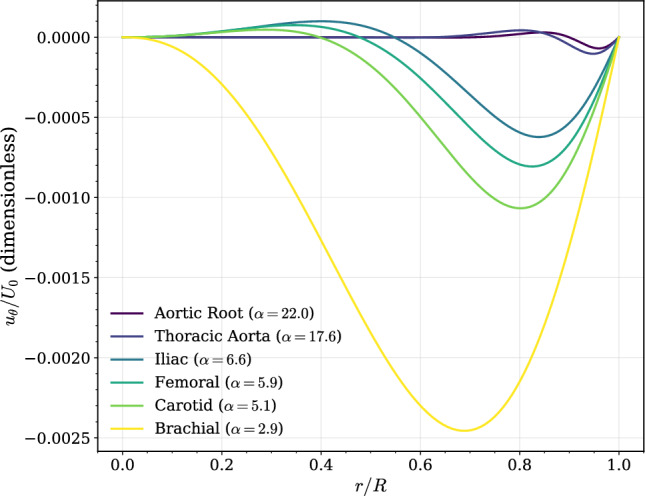
Azimuthal velocity profiles at peak systole for representative arterial waveforms. Profiles show the normalized azimuthal velocity $u_\theta /U_0$ as a function of the normalized radius *r*/*R* for different vessels, ordered by decreasing Womersley number $\alpha$. Physiological specificity refers to the harmonic content of the imposed pressure-gradient waveforms; the underlying flow model remains analytical and is based on straight-vessel anisotropic Womersley flow.

Figure 4 shows the dimensionless swirl velocity profiles at the moment of peak systole for a fixed anisotropy of $\beta =0.1$. The results show a clear distinction between high-$\alpha$ and low-$\alpha$ arteries. In large, inertia-dominated arteries like the Aortic Root ($\alpha =22.0$) and Thoracic Aorta ($\alpha =17.6$), the swirl profile is blunt and confined to a thin layer near the wall. In smaller, more viscous-dominated arteries like the Brachial ($\alpha =2.9$), the swirl profile is smoother and penetrates more deeply towards the vessel core, though its magnitude is larger.

**Fig. 5:**
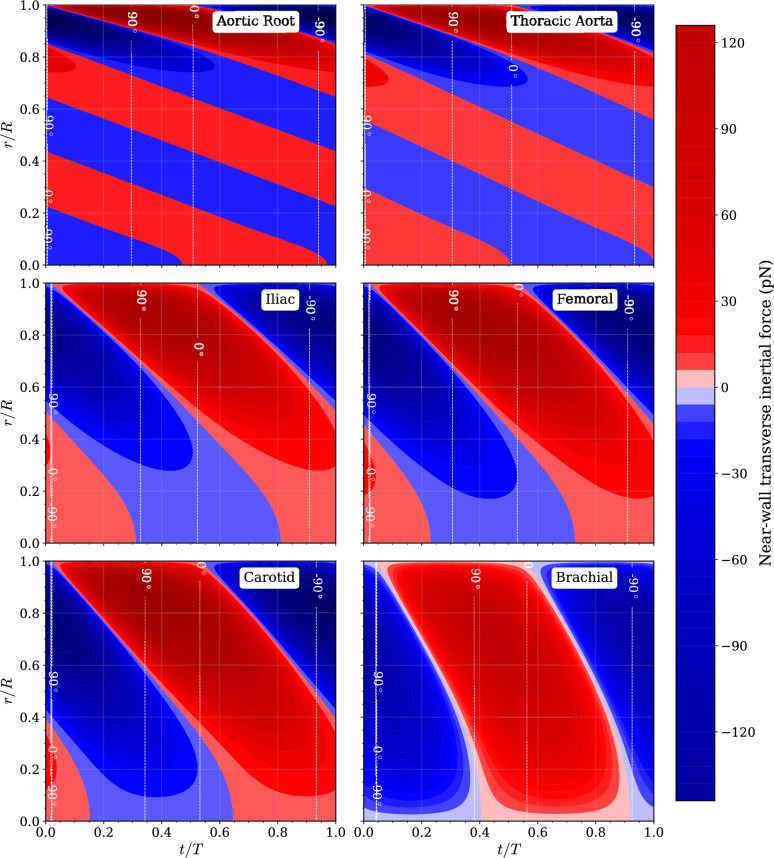
Spatiotemporal structure of the transverse inertial force field over the cardiac cycle at anisotropy ratio $\beta = 0.1$. Colour maps show the signed local near-wall transverse inertial force, derived from the Lamb vector $u \times \omega$ and evaluated adjacent to the vessel wall, with positive values indicating outward-directed forces and negative values indicating inward-directed forces. The diverging colour scale is symmetric about zero. Dashed contours indicate the instantaneous angle of the total shear vector relative to the axial direction.

To characterize the nature of the transverse force, Fig. 5 presents spatiotemporal heatmaps of the radial force field, $f_r(r,t)$, for all six arteries at a representative anisotropy of $\beta =0.1$. The colour indicates the magnitude and direction of the force (red for outward, blue for inward), while the overlaid dashed contour lines show the angle of the total hemodynamic force vector (radial force vs. axial shear force) at the near-wall location.

The results reveal a profoundly different spatiotemporal structure of the force field in large versus small arteries. In the Aortic Root ($\alpha =22.0$), the force field is complex, with multiple sign changes across the radius and throughout the cardiac cycle. The force vector angle at the wall is highly dynamic, rapidly oscillating between positive and negative values. In contrast, in the Brachial artery ($\alpha =2.9$), the force field is much simpler, with a single, dominant inward-acting phase during systole that penetrates the entire vessel. The force vector angle at the wall remains largely negative, indicating a persistent directional signal. This demonstrates that the character of the anisotropic force—its magnitude, direction, and temporal complexity—is highly dependent on the local physiological environment.

**Fig. 6:**
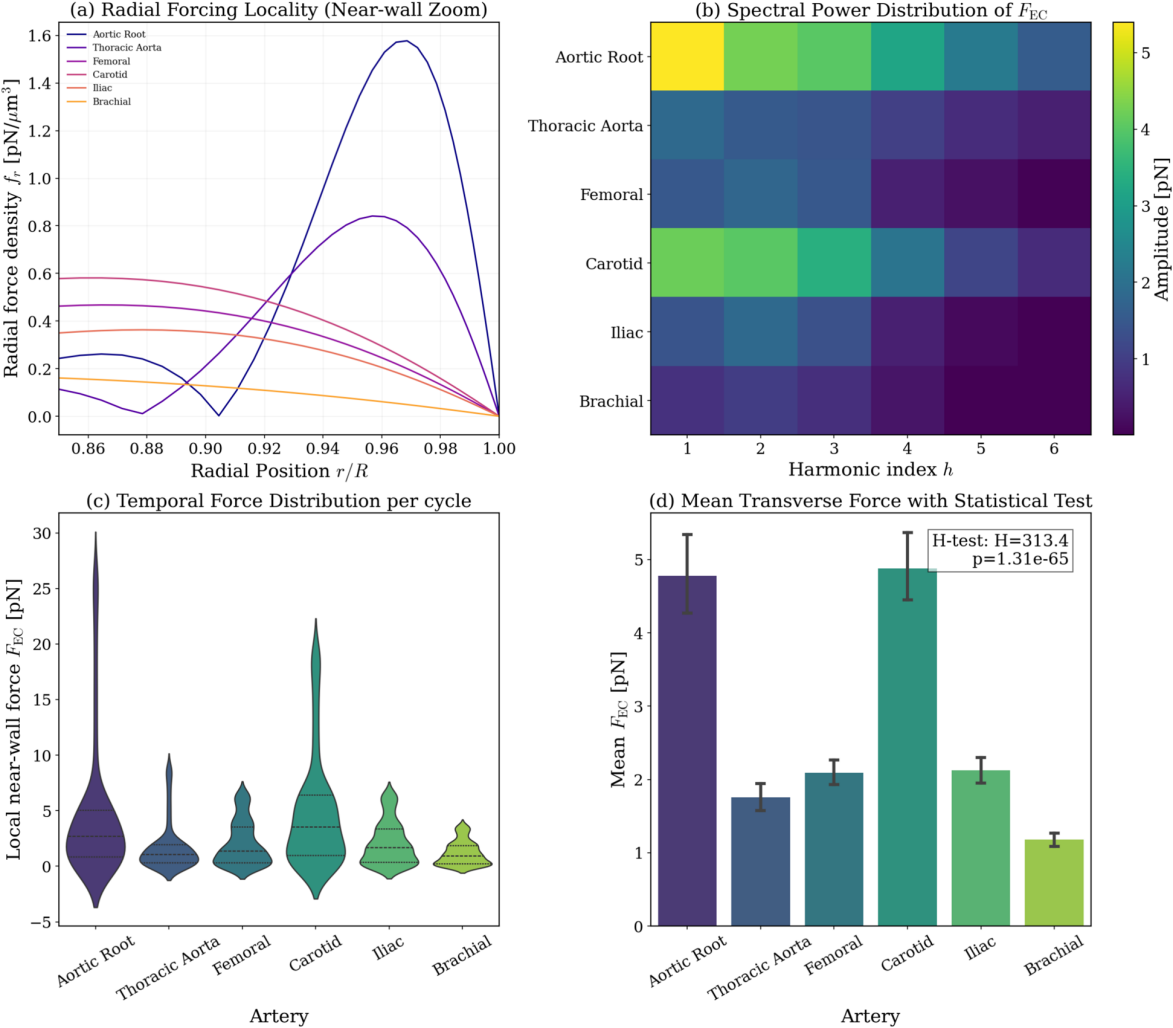
Comprehensive control volume analysis of endothelial loading at anisotropy ratio $\beta = 0.1$. (**a**) Radial profile of the transverse inertial force density at peak systole, illustrating the extreme near-wall locality. (**b**) Spectral power distribution (heatmap) of the integrated force $F_{\textrm{EC}}$ across the first six harmonics, revealing nonlinear spectral redistributions in high-$\alpha$ vessels. (**c**) Violin plots showing the temporal distribution of $F_{\textrm{EC}}$ over the cardiac cycle. (**d**) Mean $F_{\textrm{EC}}$ across the arterial tree, validated by a Kruskal–Wallis H-test confirming statistically distinct forcing regimes ($p < 10^{-4}$).

To rigorously quantify the cumulative effect of these complex spatiotemporal fields on the endothelial boundary, the force density was integrated over a near-wall cellular control volume (Fig. 6). Panel (a) isolates the radial profile of the forcing at peak systole, demonstrating the extreme spatial locality of the inertial gradients; the force density remains negligible in the core and sharply escalates exclusively within the near-wall oscillatory boundary layer ($r/R > 0.85$).

Spectral decomposition of the integrated force $F_{\textrm{EC}}$ (Fig. 6b) reveals the profound impact of nonlinear velocity-vorticity coupling. While the driving pressure waveforms are dominated by the fundamental harmonic, the nonlinear Lamb vector redistributes kinetic energy into higher-order harmonics. This frequency-doubling cascade is particularly severe in large, high-$\alpha$ vessels like the aortic root, producing a distinct, high-frequency spectral footprint.

The temporal distribution of the integrated picoNewton forces across the cardiac cycle is captured in Fig. 6c. High-$\alpha$ arteries exhibit broad, multi-modal distributions reflective of their rapid sign reversals, whereas low-$\alpha$ vessels like the brachial artery show a highly concentrated, persistent forcing magnitude. The mean integrated forces (Fig. 6d) scale systematically with vessel size. A Kruskal–Wallis H-test confirms that the anatomical variation in these inertial loads is statistically robust ($p < 10^{-4}$), demonstrating that the anisotropic transverse forcing is uniquely and distinctly tuned to the local physiological environment.

### Relative magnitude and directional modulation of the transverse force

To quantify the transverse-to-axial balance underlying the spatiotemporal patterns in Fig. 5, the local near-wall radial inertial force proxy, $F_r^{\textrm{wall}}(t)$, derived from the Lamb-vector density $f_r=\rho \,\ell _r$ and evaluated adjacent to $r=R$, is compared with the corresponding axial shear-force proxy $F_z^{\textrm{wall}}(t)=|\tau _w(t)|A_{\textrm{ref}}$, where $A_{\textrm{ref}}$ is a fixed near-wall reference area used solely for consistent magnitude scaling. Their instantaneous ratio and vector angle are defined as

$$\chi (t)=\frac{|F_r^{\textrm{wall}}(t)|}{|F_z^{\textrm{wall}}(t)|}, \qquad \varphi (t)=\tan ^{-1}\!\left( \frac{F_r^{\textrm{wall}}(t)}{F_z^{\textrm{wall}}(t)}\right) .$$

For representative arterial shear stresses of 1–$3~\textrm{Pa}$ and a reference near-wall area $A_{\textrm{ref}}\sim 100~\upmu \textrm{m}^2$, the axial force scale is $F_z^{\textrm{wall}}\sim 100$–$300~\textrm{pN}$. The anisotropy-induced radial contribution reported in Fig. 6 spans 0.1–$7~\textrm{pN}$ across vessels and anisotropy ratios, yielding a typical amplitude ratio $\chi \sim 10^{-2}$–$10^{-1}$ during phases of pronounced shear. Although the radial component is smaller in magnitude, it exerts a disproportionately strong influence on *direction*. This is visible in Fig. 5: the dashed contours show that the force-vector angle $\varphi (t)$ frequently undergoes excursions of tens of degrees and, in intermediate-$\alpha$ arteries (Iliac, Femoral, Carotid), sustained rotations approaching or exceeding $90^\circ$. In high-$\alpha$ vessels (Aortic Root, Thoracic Aorta), rapid sign changes in $F_r^{\textrm{wall}}(t)$ during brief intervals of reduced axial shear generate sharp angular swings, while in low-$\alpha$ arteries (Brachial) the radial component imposes a persistent oblique orientation throughout systole. These behaviors arise because the axial component passes through minima and reversals over the cardiac cycle, whereas the anisotropy-driven radial forcing need not vanish at the same instants.

Thus, the transverse inertial contribution dynamically modulates the *orientation* and temporal structure of the total near-wall force vector, producing directional signals that are vessel-specific and potentially relevant to endothelial-scale forcing.

### Frequency-dependent constitutive versus geometric dominance

To evaluate the relative dominance of the anisotropic Lamb vector against curvature-driven secondary flows, the bulk geometric driver $F_{c,\textrm{EC}}$ was computed using the conservative upper-bound estimate introduced in the Methods. The macroscopic bulk velocity was used to capture the maximum plausible curvature-driven forcing, scaled by the geometric factor $(1+\kappa )$ where $\kappa = 0.35 - 0.70$ for the Aortic Root, $0.20 - 0.40$ for the Thoracic Aorta, and $0.10 - 0.60$ for the Carotid artery. Following the first-order inertial response estimate summarized in Supplementary Note SI1, Eqs. (SI23)–(SI24), the resulting geometric spectrum was then attenuated by the corresponding 1/*h* factor.

**Fig. 7:**
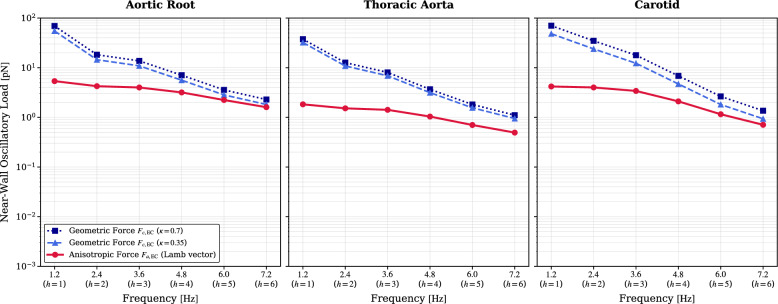
Frequency-dependent dominance analysis comparing the constitutive anisotropic cascade against the inertially filtered geometric driver. The bulk geometric force $F_{c,\textrm{EC}}$ (blue lines) was calculated using the macroscopic bulk velocity scaled by the extreme and standard curvature ratios $(\kappa )$, and subjected to the theoretical 1/*h* inertial low-pass filter to represent the suppression of the bulk curvature-driven response at higher harmonics. The anisotropic Gromeka-Lamb force $F_{a,\textrm{EC}}$ (red line) is evaluated strictly from near-wall gradients. While the geometric force dominates at the fundamental frequency ($h=1$), bulk fluid inertia dictates a total spectral collapse at higher harmonics. The anisotropic force evades this damping, emerging as the dominant mechanical stimulus for high-frequency mechanotransduction ($h \ge 3$).

Figure 7 presents the resulting spectral landscape comparing this inertially filtered geometric driver against the near-wall anisotropic force $F_{a,\textrm{EC}}$. At the fundamental cardiac frequency ($h=1$, $\sim 1.2$ Hz), the bulk geometric force dominates the transverse loading by approximately an order of magnitude. However, as the harmonic index increases, the native inertia of the bulk fluid column forces the geometric driver to undergo a rapid spectral collapse. By $h \ge 3$, the geometry-driven loads attenuate significantly. In stark contrast, the anisotropic Gromeka-Lamb force—which is constitutively driven by sharp, near-wall gradients that evade macroscopic inertial damping—maintains a persistent spectral signature. This creates a distinct frequency-dependent dominance, establishing anisotropic viscosity as the exclusive driver of transverse mechanotransduction at higher frequencies.

## Discussion

Introducing a tensorial viscosity into the Navier–Stokes equations expands the classical Womersley framework by permitting dynamics that are identically forbidden under isotropy. The results establish a consistent mechanistic sequence: anisotropic shear transport generates azimuthal velocity, this swirl field produces axial vorticity, and the interaction $\boldsymbol{\ell }=\mathbf{u}\times \boldsymbol{\omega }$ yields a transverse inertial force of picoNewton magnitude. This pathway operates in a purely axial, straight-tube geometry and therefore demonstrates that viscosity anisotropy alone is sufficient to generate multidirectional near-wall forcing.

A key structural property of this mechanism is its spectral character. Because the governing equations are linear in the velocity field for each harmonic, azimuthal velocity and axial vorticity are generated at the same discrete frequencies $\omega _h$ as the imposed pressure-gradient harmonics. The radial Lamb-vector component,

$$\ell _r = u_\theta \,\omega _z - u_z\,\omega _\theta ,$$

is therefore spectrally supported exclusively on the cardiac harmonics of the driving waveform. Its spectral carrier is constitutive and waveform-determined. By contrast, curvature-driven secondary flows arise from centrifugal acceleration embedded in $(\mathbf{u}\cdot \nabla )\mathbf{u}$ and are governed by the Dean number $\textrm{De}$^34,35^. Curvature enters through the geometric ratio $R/\mathcal {R}$ and generates three-dimensional velocity fields even in isotropic fluids^36,37^. The two mechanisms therefore occupy independent parameter directions: anisotropy through the viscosity-tensor ratios $(\beta ,\gamma ,\delta )$, and curvature through geometry-dependent convective acceleration. In the straight-tube limit ($\mathcal {R}\rightarrow \infty$, $\textrm{De}=0$), curvature contributions vanish identically while the anisotropic mechanism remains fully active.

A scaling comparison clarifies the relative magnitude of the constitutive and geometric mechanisms at the fundamental flow scale. For this purpose, the conservative geometric estimate introduced in Eq. (SI17) gives the characteristic curvature-driven transverse acceleration scale as $a_c = O\!\left( U^2(1+\kappa )/R\right)$, with $\kappa =R/\mathcal {R}$. For a highly anisotropic fluid ($\beta \sim 0.1$) in a tightly curved artery ($\kappa = R/\mathcal {R} \sim 0.3$)^38,39^, the ratio of the anisotropic acceleration to the geometric acceleration yields $a_a/a_c \sim 0.07$. This quasi-steady scaling confirms that at the fundamental cardiac frequency ($h=1$), the transverse force due to macroscopic curvature is roughly an order of magnitude larger than that generated by anisotropic viscosity, as correctly reflected in the low-frequency domain of Fig. 7.

However, this quasi-steady dominance does not hold across the broadband physiological spectrum. As clarified in Supplementary Note SI1, Eqs. (SI23)–(SI24), the bulk curvature-driven response carries a first-order inertial attenuation factor proportional to 1/*h*, so its contribution undergoes a progressive spectral collapse as harmonic number increases. By contrast, the anisotropic near-wall force is evaluated directly from the computed velocity–vorticity fields, and its interpretation is tied only to the standard harmonic Womersley scaling $\delta _{\textrm{W},h}=O(h^{-1/2})$ given by Eqs. (SI20)–(SI22). Therefore, while curvature dictates the low-frequency bulk flow and the primary temporal orientation of the force vector, the anisotropic mechanism remains the dominant source of the high-frequency near-wall transverse signal shown in Fig. 7.

Directionality further distinguishes the mechanism. Under the imposed symmetry constraints ($u_r=0$, $\partial _\theta =0$), the Lamb vector reduces to a single radial component, $\boldsymbol{\ell } = \ell _r(r,t)\,\mathbf{e}_r$, so that the anisotropy-induced inertial forcing is strictly normal to the wall in the canonical Womersley limit. Curvature-driven secondary flows, in contrast, generate three-dimensional velocity and vorticity fields at the wall and therefore produce inertial loading that is not purely wall-normal. The present formulation therefore isolates a constitutive transverse mode whose leading-order direction is wall-normal and independent of geometric torsion.

The mono-harmonic analysis (Figs. 2 and 3) clarifies the governing scalings. The linear dependence of $|\hat{U}_\theta |$ on $\beta$ confirms that off-diagonal viscosity terms are the exclusive source of swirl in this geometry. The non-monotonic dependence on $\alpha$ reflects competition between transient inertia and anisotropic diffusion, with maximal azimuthal response at intermediate Womersley numbers. In contrast, the transverse Lamb-vector force increases monotonically with $\alpha$ (Fig. 2d) because higher frequencies steepen near-wall gradients in both $u_z$ and $u_\theta$. Consequently, even modest anisotropy produces mechanically significant transverse inertia when the oscillatory boundary layer becomes thin.

Extension to physiological waveforms (Figs. 4, 5, 6) demonstrates that this force is vessel-dependent in both magnitude and temporal structure. High-$\alpha$ arteries exhibit swirl confined to a narrow oscillatory boundary layer (Fig. 4), producing transverse forces of several picoNewtons (Fig. 6d). These magnitudes overlap with forces required to gate mechanosensitive ion channels and adhesion molecules^40–43^. Lower-$\alpha$ arteries generate smaller forces but display deeper penetration and smoother temporal evolution. The spatiotemporal maps (Fig. 5) show that large arteries experience rapid sign reversals and multi-axial modulation, whereas smaller arteries exhibit more coherent inward-directed phases. Thus, anisotropy embeds frequency-dependent heterogeneity directly into pulsatile near-wall mechanics.

The implementation of a near-wall control volume integration further solidifies the physical relevance of this transverse mode. By shifting from a pointwise evaluation to a cumulative radial integration across the endothelial boundary layer, the model directly addresses the momentum flux transmitted from the fluid domain to the cell surface. Crucially, the nonlinear interaction of the physical fields within this volume generates a distinct spectral signature. As shown in the spectral heatmaps (Fig. 6b), the Lamb-vector coupling acts as a nonlinear spectral redistribution, transferring power from the fundamental heart rate into higher-order harmonics. Consequently, the endothelium is subjected to high-frequency transverse oscillatory loading that is entirely invisible to linear WSS calculations. Because mechanosensitive ion channels (e.g., Piezo1) and focal adhesions exhibit frequency-dependent activation thresholds, this anisotropy-driven spectral cascade provides a plausible, purely fluid-dynamic mechanism for the differential mechanotransductive signalling observed across varying arterial beds.

This mechanism is conceptually distinct from multidirectional shear metrics used in atherosclerosis research^3,44^. Multidirectional shear quantifies variation of surface traction arising from disturbed or geometrically complex flow. Here, even in a straight, axisymmetric vessel, anisotropic viscosity produces an intrinsic volumetric source of multidirectionality through $\boldsymbol{\ell } = \mathbf{u}\times \boldsymbol{\omega }$, which appears explicitly in the governing equations^26,27^. Because WSS is a boundary projection of the stress tensor^9,21^, it cannot capture this velocity–vorticity interaction. The Lamb vector therefore provides a mechanistically grounded near-wall descriptor that precedes the traction ultimately transmitted to the endothelium.

A plausible physiological origin of the anisotropy represented here is the Red Blood Cell Free Layer (CFL), a plasma-rich region adjacent to the wall formed by shear-induced migration of deformable red blood cells^45^. Microstructural organization within this layer generates direction-dependent stress components and off-diagonal terms in the bulk stress tensor^46,47^. Although the present model employs an effective continuum tensor rather than resolving CFL dynamics explicitly, evaluation of $\boldsymbol{\ell }$ at near-wall collocation points naturally reflects the region where such anisotropy is strongest.

The formulation remains intentionally minimal: a rigid, straight vessel and a linear anisotropic Newtonian constitutive law. Real blood exhibits shear-dependent viscosity, viscoelasticity, and time-dependent microstructural rearrangement^19,30^. These effects introduce memory and nonlinear rheology not captured by constant viscosity coefficients. Nevertheless, simulations and experiments consistently demonstrate that RBC deformation and alignment generate anisotropic stress components^17,31^. The present solution isolates the fundamental inertial mode arising from this macroscopic anisotropy in its analytically tractable form. The anisotropic Womersley solution identifies a geometry-independent, constitutive transverse inertial mode within the straight-tube Womersley limit. More complex geometry or rheology may alter its magnitude and relative importance, so its in vivo role remains to be quantified in curved and patient-specific settings. The present results therefore provide a proof-of-principle framework for incorporating viscosity anisotropy into future models of pulsatile hemodynamics and endothelial-scale mechanobiology.

## Conclusion

The anisotropic extension of the classical Womersley problem developed here demonstrates that viscosity anisotropy fundamentally modifies the inertial structure of pulsatile flow in a straight, axisymmetric vessel. By shifting the analysis from pointwise evaluation to a cumulative control-volume integration, this study establishes that constitutive coupling generates vessel-dependent transverse forcing that is statistically distinct across the arterial tree. This mechanism reveals a frequency-dependent shift in the dominant transverse forcing drivers. While macroscopic geometric drivers dominate the fundamental cardiac frequency, their magnitude is suppressed at higher harmonics due to the massive inertia of the bulk fluid column. The anisotropic Lamb vector remains mechanically relevant in this higher-frequency bandwidth, as the sharpening of near-wall gradients at high Womersley numbers counteracts macroscopic inertial attenuation. This discovery provides a controlled reference framework for separating geometric and constitutive effects in arterial hemodynamics and establishes a rigorous basis for incorporating direction-dependent blood rheology into the next generation of patient-specific vascular models.

## Supplementary Information

Supplementary Information.

## Data Availability

The code used in this study is made publicly available on: https://github.com/khalid-saqr/picoNewton/

## Abbreviations

CFD: Computational Fluid Dynamics (–)
WSS: Wall Shear Stress (–)
EC: Endothelial Cell (–)
CFL: Red Blood Cell-Free Layer (–)

## Roman symbols

$a_h$: Dimensionless pressure-gradient amplitude for harmonic *h* (–)
$A_{\textrm{EC}}$: Endothelial cell footprint area (m$^{2}$)
$F_{\textrm{EC}}$: Local near-wall inertial force per endothelial cell (N)
$F_{r,\textrm{EC}}$: Radial inertial force per endothelial cell (N)
$F_{z,\textrm{EC}}$: Axial shear force per endothelial cell (N)
$\mathbf{f}$: Inertial force density, $\mathbf{f}=\rho \,\boldsymbol{\ell }$ (N m$^{-3}$)
$f_r$: Radial component of inertial force density (N m$^{-3}$)
$f_h$: Dimensionless harmonic frequency ($f_h=\omega _h/\omega _0=h$) (–)
$\hat{G}_h$: Complex amplitude of pressure gradient (harmonic *h*) (Pa m$^{-1}$)
$h,\,H$: Harmonic index; maximum harmonic retained (–)
$L_0,\,L_1$: Linear differential operators in cylindrical coordinates (–)
*N*: Number of Chebyshev–Gauss–Lobatto collocation nodes (–)
*p*: Pressure (Pa)
*r*: Radial coordinate (m)
*R*: Vessel radius (m)
$\mathcal {R}$: Radius of curvature of vessel centerline (m)
*t*: Time (s)
$u_r,\,u_\theta ,\,u_z$: Velocity components (radial, azimuthal, axial) (m s$^{-1}$)
$\hat{u}_{j,h}$: Complex velocity amplitude of component *j* at harmonic *h* (m s$^{-1}$)
$U_0$: Characteristic velocity scale (m s$^{-1}$)
$V_{\textrm{EC}}$: Endothelial cell control volume (m$^{3}$)
*z*: Axial coordinate (m)
$$\chi (t)$$: Instantaneous ratio $$|F_{r,\textrm{EC}}|/|F_{z,\textrm{EC}}|$$ (–)
$\varphi (t)$: Instantaneous force-vector angle at the wall (rad)

## Greek symbols

$$\alpha$$: Womersley number, $$R\sqrt{\omega _0/\nu _{zz}}$$ (–)
$$\beta$$: Anisotropy ratio, $$\nu _{z\theta }/\nu _{zz}$$ (–)
$$\gamma$$: Anisotropy ratio, $$\nu _{\theta z}/\nu _{zz}$$ (–)
$$\delta$$: Anisotropy ratio, $$\nu _{\theta \theta }/\nu _{zz}$$ (–)
$\delta _{\textrm{W}}$: Oscillatory (Womersley) boundary-layer thickness (m)
$$\boldsymbol{\ell }$$: Lamb vector, $$\boldsymbol{\ell }=\mathbf{u}\times \boldsymbol{\omega }$$ (m s$$^{-2}$$)
$\ell _r$: Radial component of Lamb vector (m s$^{-2}$)
$\nu _{ij}$: Components of kinematic viscosity tensor (m$^{2}$ s$^{-1}$)
$\rho$: Fluid density (kg m$^{-3}$)
$\tau _{ij}$: Components of shear stress tensor Pa
$\theta$: Azimuthal coordinate (rad)
$\omega _0$: Fundamental angular frequency (rad s$^{-1}$)
$\omega _h$: Angular frequency of harmonic *h* (rad s$^{-1}$)
$$\boldsymbol{\omega }$$: Vorticity vector (s$$^{-1}$$)
$\omega _\theta ,\,\omega _z$: Vorticity components (azimuthal, axial) (s$^{-1}$)
$\textrm{De}$: Dean number (curvature parameter) (–)

## Subscripts and superscripts

^: Complex amplitude (frequency-domain quantity) (–)
$$^*$$: Dimensionless quantity (–)
$_0$: Fundamental harmonic or characteristic scale (–)
$_{\textrm{EC}}$: Quantity evaluated at endothelial scale (–)
$_h$: Quantity associated with *h*-th harmonic (–)
$_r,\,_\theta ,\,_z$: Cylindrical components (radial, azimuthal, axial) (–)

## Footnotes

1 The complete open-source Python code, including the solver and scripts to reproduce all figures, is available at https://github.com/khalid-saqr/picoNewton.
