# Supplementary material for: A transverse picoNewton force revealed in anisotropic Womersley flow
Source: Sci Rep. 2026 Apr 16;16:12584. doi: 10.1038/s41598-026-47474-x (PMC13087026; doi:10.1038/s41598-026-47474-x)
Supplement: Supplementary file 1 — Supplementary Information. [file 41598_2026_47474_MOESM1_ESM.pdf]

Supplementary Information Notes

A transverse picoNewton force revealed in anisotropic Womersley flow

Khalid M. Saqr

Mechanical Engineering Department, College of Engineering and Technology
Arab Academy for Science, Technology, and Maritime Transport, Alexandria, Egypt
ORCID: 0000-0002-3058-2705 Email: k.saqr@aast.edu

Table of Contents

This supplementary file contains four notes. The list below provides a brief description of each note and its starting page.

1. **Note SI1: Derivation of the Anisotropic Womersley Equations** — detailed derivation from incompressible Navier–Stokes to the coupled anisotropic frequency-domain boundary-value problem. . . . . p. S2

2. **Note SI2: Relation between the flow and the force transmitted to the wall** — control-volume and Gromeka–Lamb formulations linking flow fields to the near-wall force balance and endothelial-scale inertial metrics. . . . . p. S7

3. **Note SI3: Spectral Collocation Method and Numerical Implementation** — Chebyshev–Gauss–Lobatto discretization, matrix assembly, boundary-condition enforcement, and sparse linear solves for each harmonic. . . . . p. S10

4. **Note SI4: Detailed Grid-Independence Verification** — successive-refinement convergence analysis confirming spectral accuracy and numerical independence of reported results. . . . . p. S13

SI1 Derivation of the Anisotropic Womersley Equations

This supplementary note provides a pedagogical, step-by-step derivation of the model's governing equations, ensuring every assumption and mathematical step is traceable.

Governing Equations

Our starting point is the universal vector form of the Navier-Stokes equations for an incompressible fluid of constant density, ρ.

The conservation of momentum is given by the Cauchy momentum equation:

$$\rho \left(\frac{\partial \mathbf{u}}{\partial t} + (\mathbf{u} \cdot \nabla)\mathbf{u} \right) = -\nabla p + \nabla \cdot \boldsymbol{\tau} \tag{SI1}$$

The conservation of mass for an incompressible fluid is given by the continuity equation:

$$\nabla \cdot \mathbf{u} = 0 \tag{SI2}$$

where $\mathbf{u}$ is the velocity vector, p is the pressure, and $\boldsymbol{\tau}$ is the deviatoric stress tensor.

Specialization for Axisymmetric Flow in a Cylindrical Vessel

We specialize these laws for a cylindrical coordinate system (r, θ, z) with velocity $\mathbf{u} = (u_r, u_\theta, u_z)$, using the following standard assumptions:

1. **Axisymmetric Flow:** $\partial/\partial\theta = 0$ for all quantities.
2. **No Radial Velocity:** $u_r = 0$ everywhere.

Applying these to the continuity equation, $\frac{1}{r}\frac{\partial}{\partial r}(ru_r) + \frac{1}{r}\frac{\partial u_\theta}{\partial \theta} + \frac{\partial u_z}{\partial z} = 0$, yields:

$$\frac{1}{r}\frac{\partial}{\partial r}(r \cdot \underbrace{0}_{u_r=0}) + \frac{1}{r}\underbrace{\frac{\partial u_\theta}{\partial \theta}}_{=0} + \frac{\partial u_z}{\partial z} = 0 \quad \Longrightarrow \quad \frac{\partial u_z}{\partial z} = 0 \tag{SI3}$$

This confirms the flow is fully developed. These assumptions cause a profound simplification of the nonlinear convective acceleration term, $(\mathbf{u} \cdot \nabla)\mathbf{u}$, as the operator itself effectively becomes zero when applied to the velocity components:

$$(\mathbf{u} \cdot \nabla) = \left(\underbrace{u_r}_{=0}\frac{\partial}{\partial r} + \frac{u_\theta}{r}\cancelto{0}{\frac{\partial}{\partial \theta}} + u_z \cancelto{0}{\frac{\partial}{\partial z}} \right) \to 0 \tag{SI4}$$

The momentum equations therefore simplify to a linear system:

$$\rho \frac{\partial u_z}{\partial t} = -\frac{\partial p}{\partial z} + \frac{1}{r}\frac{\partial}{\partial r}(r\tau_{zr}) \tag{SI5}$$

$$\rho \frac{\partial u_\theta}{\partial t} = \frac{1}{r^2}\frac{\partial}{\partial r}(r^2\tau_{\theta r}) \tag{SI6}$$

Deriving the Anisotropic Constitutive Relations

The physics of the fluid are defined by the relationship between the deviatoric stress tensor, $\boldsymbol{\tau}$, and the rate-of-strain tensor, $\mathbf{S} = \frac{1}{2}(\nabla\mathbf{u} + (\nabla\mathbf{u})^T)$. For a general linear (Newtonian) anisotropic fluid, this is a contraction of a fourth-rank viscosity tensor $\boldsymbol{\mathcal{C}}$ with the strain rate: $\tau_{ij} = \mathcal{C}_{ijkl}S_{kl}$.

First, we determine the non-zero components of the strain rate tensor $\mathbf{S}$ under our flow assumptions ($u_r = 0$, $\partial_\theta = 0$, $\partial_z\mathbf{u} = 0$). In cylindrical coordinates, the only surviving terms are the shear rates acting on planes perpendicular to the radial direction:

$$S_{zr} = S_{rz} = \frac{1}{2}\left(\frac{\partial u_z}{\partial r} + \underbrace{\frac{\partial u_r}{\partial z}}_{0}\right) = \frac{1}{2}\frac{\partial u_z}{\partial r} \tag{SI7}$$

$$S_{\theta r} = S_{r\theta} = \frac{1}{2}\left(r\frac{\partial}{\partial r}\left(\frac{u_\theta}{r}\right) + \underbrace{\frac{1}{r}\frac{\partial u_r}{\partial \theta}}_{0}\right) = \frac{1}{2}\left(\frac{\partial u_\theta}{\partial r} - \frac{u_\theta}{r}\right) \tag{SI8}$$

All other components ($S_{rr}, S_{\theta\theta}, S_{zz}, S_{z\theta}$) represent elongational rates or shears not present in this flow geometry and are zero.

The general constitutive law thus reduces to relations for the relevant shear stresses, τ_{zr} and $\tau_{\theta r}$. The anisotropy implies that a strain rate in one direction can generate stress in another. Defining kinematic viscosities $\nu = \mathcal{C}/\rho$ (absorbing factors of 1/2), we arrive at the coupled system:

$$\begin{bmatrix}\tau_{zr}\\ \tau_{\theta r}\end{bmatrix} = \rho\begin{bmatrix}\nu_{zz} & \nu_{z\theta}\\ \nu_{\theta z} & \nu_{\theta\theta}\end{bmatrix}\begin{bmatrix}2S_{zr}\\ 2S_{\theta r}\end{bmatrix} = \rho\begin{bmatrix}\nu_{zz} & \nu_{z\theta}\\ \nu_{\theta z} & \nu_{\theta\theta}\end{bmatrix}\begin{bmatrix}\partial_r u_z\\ \partial_r u_\theta - u_\theta/r\end{bmatrix} \tag{SI9}$$

This 2×2 matrix is the specific realization of the general fourth-rank tensor for this restricted flow geometry. The off-diagonal terms $\nu_{z\theta}$ and $\nu_{\theta z}$ are explicitly responsible for the cross-coupling.

Substitution and Transformation to the Frequency Domain

The governing PDEs are linear. We exploit this by decomposing the driving pressure gradient and the resulting velocities into Fourier series. This transforms the problem from solving PDEs in time t to solving ODEs for the complex amplitudes of each harmonic frequency ω_h:

$$-\frac{\partial p}{\partial z}(t) = \sum_h \hat{G}_h e^{i\omega_h t}, \quad u_z(r,t) = \sum_h \hat{u}_{z,h}(r)e^{i\omega_h t}, \quad u_\theta(r,t) = \sum_h \hat{u}_{\theta,h}(r)e^{i\omega_h t}$$

Consider a single harmonic h (dropping the subscript for clarity). The time derivatives become $\frac{\partial u}{\partial t} = i\omega\hat{u}e^{i\omega t}$.

We now substitute the constitutive expressions from Eq. (SI9) into the time-domain momentum equations and cancel the common $e^{i\omega t}$ and density ρ terms.

For the **z-momentum** equation:

$$i\omega\hat{u}_z = \frac{\hat{G}}{\rho} + \frac{1}{r}\frac{d}{dr}\left(r\left[\nu_{zz}\frac{d\hat{u}_z}{dr} + \nu_{z\theta}\left(\frac{d\hat{u}_\theta}{dr} - \frac{\hat{u}_\theta}{r}\right)\right]\right)$$

Applying the product rule $\frac{1}{r}\frac{d}{dr}(rA) = \frac{dA}{dr} + \frac{A}{r}$ to the terms on the right-hand side:

$$i\omega\hat{u}_z = \frac{\hat{G}}{\rho} + \nu_{zz}\underbrace{\left(\frac{d^2\hat{u}_z}{dr^2} + \frac{1}{r}\frac{d\hat{u}_z}{dr}\right)}_{\text{Defines operator } L_0\hat{u}_z} + \nu_{z\theta}\underbrace{\left(\frac{d}{dr}\left(\frac{d\hat{u}_\theta}{dr} - \frac{\hat{u}_\theta}{r}\right) + \frac{1}{r}\left(\frac{d\hat{u}_\theta}{dr} - \frac{\hat{u}_\theta}{r}\right)\right)}_{\text{Defines operator } L_1\hat{u}_\theta}$$

Evaluating the derivative in the second bracket yields $\left(\hat{u}_\theta'' - \frac{\hat{u}_\theta'}{r} + \frac{\hat{u}_\theta}{r^2}\right) + \left(\frac{\hat{u}_\theta'}{r} - \frac{\hat{u}_\theta}{r^2}\right) = \hat{u}_\theta'' - \frac{\hat{u}_\theta'}{r} + \frac{\hat{u}_\theta}{r^2} + \frac{\hat{u}_\theta'}{r} - \frac{\hat{u}_\theta}{r^2}$. Evaluating the standard form of the vector Laplacian component for u_θ, the term identified as $L_1\hat{u}_\theta$ simplifies to $\frac{d^2\hat{u}_\theta}{dr^2} + \frac{1}{r}\frac{d\hat{u}_\theta}{dr} - \frac{\hat{u}_\theta}{r^2}$.

Similarly, for the **θ-momentum** equation:

$$i\omega\hat{u}_\theta = \frac{1}{r^2}\frac{d}{dr}\left(r^2\left[\nu_{\theta z}\frac{d\hat{u}_z}{dr} + \nu_{\theta\theta}\left(\frac{d\hat{u}_\theta}{dr} - \frac{\hat{u}_\theta}{r}\right)\right]\right)$$

Carrying out the differentiation $\frac{1}{r^2}\frac{d}{dr}(r^2A) = \frac{dA}{dr} + \frac{2A}{r}$ reveals the same operators L_0 acting on the axial part and L_1 on the azimuthal part.

Thus, we arrive at the coupled ODE system for the amplitudes:

$$i\omega\hat{u}_z = \frac{\hat{G}}{\rho} + \nu_{zz}L_0\hat{u}_z + \nu_{z\theta}L_1\hat{u}_\theta \tag{SI10}$$

$$i\omega\hat{u}_\theta = \nu_{\theta z}L_0\hat{u}_z + \nu_{\theta\theta}L_1\hat{u}_\theta \tag{SI11}$$

Where the operators are rigorously defined as $L_0 f = f'' + \frac{1}{r}f'$ and $L_1 f = f'' + \frac{1}{r}f' - \frac{f}{r^2}$.

Non-dimensionalization to Reveal Governing Parameters

We non-dimensionalize using the scales R, ω_0, and $U_0 = |\hat{G}_0|R^2/(\rho\nu_{zz})$, and variables $r^*, \hat{U}_j^*, a_h$. This process groups physical constants into the governing dimensionless parameters: Womersley number (α), and anisotropy ratios (β, γ, δ).

The Final Boundary Value Problem

The derivation concludes by presenting the final, well-posed mathematical problem that is passed to the numerical solver for each harmonic h:

$$if_h\alpha^2\hat{U}_z^* = a_h + L_0^*\hat{U}_z^* + \beta L_1^*\hat{U}_\theta^* \tag{SI12}$$

$$if_h\alpha^2\hat{U}_\theta^* = \gamma L_0^*\hat{U}_z^* + \delta L_1^*\hat{U}_\theta^* \tag{SI13}$$

The harmonic dependence used in the main text follows directly from the dimensionless unsteady coefficient in Eqs. (SI12)–(SI13). Using

$$f_h = \frac{\omega_h}{\omega_0}, \qquad \alpha = R\sqrt{\frac{\omega_0}{\nu_{zz}}},$$

one obtains

$$f_h\alpha^2 = \frac{\omega_h}{\omega_0}\frac{R^2\omega_0}{\nu_{zz}} = \frac{R^2\omega_h}{\nu_{zz}}. \tag{SI14}$$

This identifies the harmonic Womersley parameter as

$$\alpha_h := \sqrt{f_h}\,\alpha = R\sqrt{\frac{\omega_h}{\nu_{zz}}}. \tag{SI15}$$

Accordingly, the oscillatory Womersley/Stokes layer associated with harmonic h obeys the standard scaling

$$\delta_{\mathrm{W},h} = O\!\left(\frac{R}{\alpha_h}\right) = O\!\left(\sqrt{\frac{\nu_{zz}}{\omega_h}}\right). \tag{SI16}$$

Since $\omega_h = h\omega_0$, it follows that

$$\delta_{\mathrm{W},h} = O(h^{-1/2}). \tag{SI17}$$

Only this scaling, not a specific prefactor, is used in the main text.

This system is subject to the following boundary conditions in the dimensionless domain:

1. **No-slip at the wall ($r^* = 1$):** $\hat{U}_z^*(1) = 0$ and $\hat{U}_\theta^*(1) = 0$.
2. **Symmetry/Regularity at the centerline ($r^* = 0$):** $\frac{d\hat{U}_z^*}{dr^*}(0) = 0$ and $\hat{U}_\theta^*(0) = 0$.

Scaling estimates used in the spectral comparison

Two distinct types of scaling are used in the main-text spectral comparison and should be distinguished clearly.

First, the oscillatory near-wall length scale follows directly from the harmonic Womersley form of Eqs. (SI12)–(SI13). Since

$$f_h = \frac{\omega_h}{\omega_0}, \qquad \alpha = R\sqrt{\frac{\omega_0}{\nu_{zz}}}, \tag{SI18}$$

their product satisfies

$$f_h\alpha^2 = \frac{\omega_h}{\omega_0}\frac{R^2\omega_0}{\nu_{zz}} = \frac{R^2\omega_h}{\nu_{zz}}. \tag{SI19}$$

It is therefore natural to define the harmonic Womersley parameter

$$\alpha_h := \sqrt{f_h}\,\alpha = R\sqrt{\frac{\omega_h}{\nu_{zz}}}. \tag{SI20}$$

The standard oscillatory Womersley/Stokes layer associated with harmonic h then has the order-of-magnitude thickness

$$\delta_{\mathrm{W},h} = O\!\left(\frac{R}{\alpha_h}\right) = O\!\left(\sqrt{\frac{\nu_{zz}}{\omega_h}}\right), \tag{SI21}$$

so that, because $\omega_h = h\omega_0$,

$$\delta_{\mathrm{W},h} = O(h^{-1/2}). \tag{SI22}$$

This is the origin of the boundary-layer-thickness scaling invoked in the main text.

Second, the $1/h$ attenuation used for the geometric comparison is not a new exact solution of a Dean-flow problem; it is a first-order inertial response estimate for a bulk curvature-driven transverse velocity scale. If $\hat{f}_{r,c,h}$ denotes the harmonic forcing amplitude associated with the geometric benchmark and $\hat{u}_{s,h}$ the corresponding cross-stream velocity scale, then the leading inertial estimate is

$$\rho\, i\omega_h\, \hat{u}_{s,h} \sim \hat{f}_{r,c,h}. \tag{SI23}$$

Hence,

$$\hat{u}_{s,h} = O\left(\frac{\hat{f}_{r,c,h}}{\rho\,\omega_h}\right), \tag{SI24}$$

so the transfer magnitude from forcing to bulk cross-stream response carries a factor proportional to $1/\omega_h \sim 1/h$. This is the basis of the inertial attenuation factor used in the main-text comparison of the curvature-driven benchmark with the anisotropy-induced near-wall force.

Accordingly, the work uses Eq. (SI22) as a standard harmonic Womersley scaling for the near-wall layer, and Eqs. (SI23)–(SI24) as a conservative order-of-magnitude estimate for the bulk curvature-driven response.

On the interpretation of endothelial-scale forcing

The Lamb vector $\boldsymbol{\ell} = \mathbf{u} \times \boldsymbol{\omega}$ represents a volumetric inertial forcing density. The endothelial-scale force reported in the main text is obtained by evaluating this quantity within a near-wall control volume representative of an endothelial cell footprint. This estimate characterizes the local inertial stimulus acting within the endothelial layer and does not correspond to a radius-integrated force over the entire vessel cross-section. A full cross-sectional sector integration would yield a distinct quantity associated with global radial momentum balance; the present study focuses specifically on the local near-wall forcing scale relevant to mechanotransductive stimuli.

SI2 Relation between the flow and the force transmitted to the wall

This note clarifies the mechanical meaning of the near-wall inertial quantity reported in the main text and its relation to the force transmitted from the fluid to the endothelium.

1. Traction is the force transmitted to the wall

In continuum mechanics, the local force per unit area transmitted across a surface with unit normal $\mathbf{n}$ is the Cauchy traction

$$\mathbf{t}(\mathbf{n}) = \boldsymbol{\sigma} \cdot \mathbf{n}, \tag{SI25}$$

where $\boldsymbol{\sigma}$ is the total fluid stress tensor. Accordingly, the actual force exerted by the fluid on an endothelial patch is obtained from the traction integrated over the wall patch, not directly from a volumetric field defined inside the fluid.

Let A_{EC} denote a small endothelial footprint on the vessel wall, and let $\mathbf{n}_w$ be the outward unit normal of the fluid domain at that patch. The force exerted by the fluid on the wall patch is

$$\mathbf{F}_w(t) = -\int_{A_{\mathrm{EC}}} \boldsymbol{\sigma} \cdot \mathbf{n}_w \, dA, \tag{SI26}$$

where the minus sign accounts for the fact that $\boldsymbol{\sigma} \cdot \mathbf{n}_w$ is the traction exerted *on the fluid* by the wall, whereas $\mathbf{F}_w$ is the equal-and-opposite force exerted by the fluid *on the wall.*

2. Pillbox control volume adjacent to the wall

To relate this wall force to the near-wall flow structure, consider a thin fluid control volume V_{EC} of thickness δ_{EC} located directly above the wall patch A_{EC},

$$V_{\mathrm{EC}} = \{(r, \theta, z) : R - \delta_{\mathrm{EC}} \le r \le R,\ (\theta, z) \in A_{\mathrm{EC}}\}, \tag{SI27}$$

with boundary

$$\partial V_{\mathrm{EC}} = A_w \cup A_\delta \cup S_{\mathrm{EC}}, \tag{SI28}$$

where $A_w = A_{\mathrm{EC}}$ is the wall face, A_δ is the outer face at $r = R - \delta_{\mathrm{EC}}$, and S_{EC} is the lateral side surface.

The local Cauchy momentum equation is

$$\rho \frac{D\mathbf{u}}{Dt} = \nabla \cdot \boldsymbol{\sigma} + \rho \mathbf{b}, \tag{SI29}$$

where $\mathbf{b}$ is any body force per unit mass. Integrating Eq. (SI29) over V_{EC} gives

$$\int_{V_{\mathrm{EC}}} \rho \frac{D\mathbf{u}}{Dt} \, dV = \int_{\partial V_{\mathrm{EC}}} \boldsymbol{\sigma} \cdot \mathbf{n} \, dA + \int_{V_{\mathrm{EC}}} \rho \mathbf{b} \, dV. \tag{SI30}$$

Separating the wall face from the rest of the boundary,

$$\int_{A_w} \boldsymbol{\sigma} \cdot \mathbf{n}_w \, dA = \int_{V_{\mathrm{EC}}} \rho \frac{D\mathbf{u}}{Dt} \, dV - \int_{A_\delta \cup S_{\mathrm{EC}}} \boldsymbol{\sigma} \cdot \mathbf{n} \, dA - \int_{V_{\mathrm{EC}}} \rho \mathbf{b} \, dV. \tag{SI31}$$

Using Eq. (SI26), the force exerted by the fluid on the wall patch is therefore

$$\mathbf{F}_w(t) = -\int_{V_{\mathrm{EC}}} \rho \frac{D\mathbf{u}}{Dt}\, dV + \int_{A_\delta \cup S_{\mathrm{EC}}} \boldsymbol{\sigma}\cdot\mathbf{n}\, dA + \int_{V_{\mathrm{EC}}} \rho\mathbf{b}\, dV. \tag{SI32}$$

Equation (SI32) is the precise control-volume statement: the wall force is the cumulative result of the fluid momentum balance in the thin near-wall layer together with stresses on the other faces of that layer.

3. Role of the Lamb vector in the control-volume balance

Using the Gromeka–Lamb decomposition,

$$(\mathbf{u}\cdot\nabla)\mathbf{u} = \nabla\big(\tfrac{1}{2}|\mathbf{u}|^2\big) - \boldsymbol{\ell}, \qquad \boldsymbol{\ell} = \mathbf{u}\times\boldsymbol{\omega}, \tag{SI33}$$

the material acceleration can be written as

$$\frac{D\mathbf{u}}{Dt} = \frac{\partial\mathbf{u}}{\partial t} + \nabla\big(\tfrac{1}{2}|\mathbf{u}|^2\big) - \boldsymbol{\ell}. \tag{SI34}$$

Substituting Eq. (SI34) into Eq. (SI32) yields

$$\begin{aligned}\mathbf{F}_w(t) = &-\int_{V_{\mathrm{EC}}} \rho\frac{\partial\mathbf{u}}{\partial t}\, dV - \int_{V_{\mathrm{EC}}} \rho\nabla\big(\tfrac{1}{2}|\mathbf{u}|^2\big)\, dV + \int_{V_{\mathrm{EC}}} \rho\boldsymbol{\ell}\, dV \\ &+ \int_{A_\delta\cup S_{\mathrm{EC}}} \boldsymbol{\sigma}\cdot\mathbf{n}\, dA + \int_{V_{\mathrm{EC}}} \rho\mathbf{b}\, dV.\end{aligned} \tag{SI35}$$

Equation (SI35) shows that the volume integral of $\rho\boldsymbol{\ell}$ is not itself the total wall traction. Rather, it is one fluid-side inertial contribution within the exact wall-force balance for the thin near-wall control volume.

4. Consequence for the present straight-tube Womersley model

In the present model, the vessel is rigid and no-slip conditions are imposed at $r = R$, so that

$$u_z(R,t) = u_\theta(R,t) = 0. \tag{SI36}$$

Therefore, at the wall,

$$\boldsymbol{\ell}(R,t) = \mathbf{u}(R,t)\times\boldsymbol{\omega}(R,t) = \mathbf{0}. \tag{SI37}$$

This implies that evaluating the Lamb vector *exactly at the wall* does not provide a non-trivial measure of endothelial loading. The mechanically meaningful quantity is instead the contribution of the Lamb-vector term integrated over a thin near-wall fluid layer, as it appears in Eq. (SI35).

5. Definition of the reported near-wall inertial proxy

Motivated by Eq. (SI35), the quantity reported in the main text is interpreted as a local fluid-side inertial contribution associated with the Lamb-vector term in a thin near-wall layer of thickness δ_{EC} above the wall patch A_{EC}:

$$\mathbf{F}^{(\ell)}_{\text{EC}}(t) = \rho \int_{V_{\text{EC}}} \boldsymbol{\ell}(r,t)\, dV. \tag{SI38}$$

Its radial component is

$$F^{(\ell)}_{r,\text{EC}}(t) = \rho \int_{V_{\text{EC}}} \ell_r(r,t)\, dV. \tag{SI39}$$

For the magnitude-based estimate used in the main article,

$$F_{\text{EC}}(t) = \rho\, V_{\text{EC}}\, \langle |\boldsymbol{\ell}(r,t)| \rangle_{\text{EC}}\,, \tag{SI40}$$

where $\langle\cdot\rangle_{\text{EC}}$ denotes averaging over the thin near-wall control volume and $V_{\text{EC}} = A_{\text{EC}}\,\delta_{\text{EC}}$.

Equation (SI40) is therefore not interpreted as the total force transmitted to the endothelial cell. Rather, it is a local estimate of the fluid-side inertial forcing associated with the Lamb-vector term in the near-wall layer that contributes to the full wall-force balance Eq. (SI35).

6. Specialization to the present axisymmetric model

Under the assumptions $u_r = 0$ and $\partial_\theta(\cdot) = 0$, the Lamb vector reduces to

$$\ell_r = u_\theta \frac{1}{r}\frac{\partial}{\partial r}(r u_\theta) + u_z \frac{\partial u_z}{\partial r}, \tag{SI41}$$

so the reported near-wall inertial contribution is purely radial within the straight-tube Womersley limit:

$$F^{(\ell)}_{r,\text{EC}}(t) = \rho \int_{V_{\text{EC}}} \left[u_\theta \frac{1}{r}\frac{\partial}{\partial r}(r u_\theta) + u_z \frac{\partial u_z}{\partial r} \right] dV. \tag{SI42}$$

This quantity captures the local radial inertial organization of the fluid adjacent to the wall. A full computation of the total endothelial traction would additionally require the pressure and viscous traction terms on the wall, as well as the remaining stress contributions on A_δ and S_{EC} appearing in Eq. (SI35).

Crucially, while the radial pressure gradient must satisfy the radial momentum balance ($\partial_r p \approx \rho \ell_r$), the spectral redistribution of the Lamb-vector term remains the primary driver of high-frequency fluctuations that cannot be counterbalanced by the bulk-driven pressure field.

SI3 Spectral Collocation Method and Numerical Implementation

The governing equations derived in supplementary note SI1 form a system of coupled, complex-valued Ordinary Differential Equations (ODEs) that do not have a simple analytical solution. This appendix provides a detailed, pedagogical guide to the spectral collocation method used to solve them, explicitly linking the mathematical theory to the implementation in the accompanying Python code. The goal is to make the numerical aspects of this work fully transparent and reproducible.

Spectral methods are chosen for their exceptionally high ("spectral") accuracy, which makes them ideal for resolving the sharp velocity gradients in the near-wall boundary layers characteristic of high-Womersley-number flows. The core strategy is to transform the continuous differential problem into a discrete system of linear algebraic equations, which can then be solved efficiently.

Step 1: The Chebyshev Collocation Grid

The first step is to discretize the radial domain, $r^* \in [0, 1]$. We use the Chebyshev–Gauss–Lobatto (CGL) points, which cluster near the boundaries and are optimal for polynomial approximation. These $N + 1$ points are first defined on a reference domain $x \in [-1, 1]$ and then mapped to the physical domain.

Theory: The CGL points are the locations of the extrema of the N-th order Chebyshev polynomial:

$$x_k = \cos\left(\frac{\pi k}{N}\right), \quad k = 0, 1, \ldots, N \tag{SI43}$$

A linear mapping then transforms these points to our radial domain $r^* \in [0, 1]$:

$$r_k^* = \frac{1 - x_k}{2} \tag{SI44}$$

This places point $k = 0$ at the centerline ($x_0 = 1 \rightarrow r_0^* = 0$) and point $k = N$ at the wall ($x_N = -1 \rightarrow r_N^* = 1$).

Code Implementation: This is implemented in the `_setup_discretization` method:

```
k = np.arange(self.n); x = np.cos(np.pi * k / self.N)
...
self.r = (1.0 - x) / 2.0
```

Step 2: The Differentiation Matrix

The defining feature of the spectral method is its representation of the derivative. For a given set of CGL points, a single $(N+1) \times (N+1)$ matrix, $\mathbf{D}$, can be constructed such that the derivative of any function $f(r^*)$ at these points is found via a matrix-vector product: $\mathbf{f}' \approx \mathbf{D}\mathbf{f}$.

Theory: The entries of the Chebyshev differentiation matrix on the reference domain, $\mathbf{D}_{cheb}$, are known analytically. The code uses a standard, robust matrix-based formula to

construct it, after which it is scaled using the chain rule to apply to our physical domain. Since $r^* = (1-x)/2$, the chain rule gives:

$$\frac{d}{dr^*} = \frac{dx}{dr^*}\frac{d}{dx} = (-2)\frac{d}{dx} \quad \Longrightarrow \quad \mathbf{D} = -2\mathbf{D}_{cheb} \tag{SI45}$$

Code Implementation: The matrix $\mathbf{D}_{cheb}$ is constructed, and then scaled to create the final differentiation matrix `self.D`.

```
# ... lines to construct D_cheb based on standard formulas ...
D_cheb = (np.outer(c, 1.0/c)) / dX
D_cheb -= np.diag(np.sum(D_cheb, axis=1))
self.D = D_cheb * (-2.0)
```

Step 3: Assembling the Discrete Differential Operators

With the differentiation matrix $\mathbf{D}$, we can construct the matrix equivalents of the continuous operators L_0^* and L_1^*:

$$L_0^* f = \frac{d^2 f}{dr^{*2}} + \frac{1}{r^*}\frac{df}{dr^*} \quad \to \quad \mathbf{L_0} = \mathbf{D}^2 + \mathbf{R}^{-1}\mathbf{D} \tag{SI46}$$

$$L_1^* f = L_0^* f - \frac{f}{r^{*2}} \quad \to \quad \mathbf{L_1} = \mathbf{L_0} - \mathbf{R}^{-2} \tag{SI47}$$

where $\mathbf{D}^2 = \mathbf{D}\mathbf{D}$ is the second derivative matrix, and $\mathbf{R}^{-1}$ and $\mathbf{R}^{-2}$ are diagonal matrices containing $1/r_k^*$ and $1/r_k^{*2}$.

Code Implementation: Sparse matrices are used for efficiency. Note the direct correspondence between the math and the code.

```
invr = sp.diags(1.0 / r_safe); invr2 = sp.diags(1.0 / (r_safe**2))
Dsp, D2sp = sp.csr_matrix(self.D), sp.csr_matrix(self.D @ self.D)
self.L0 = D2sp + invr @ Dsp; self.L1 = self.L0 - invr2
```

Step 4: Constructing the Full Block-Matrix System

The two coupled ODEs are now assembled into a single $2(N+1) \times 2(N+1)$ system of linear algebraic equations, $\mathbf{A}\mathbf{x} = \mathbf{b}$.

Theory: The matrix $\mathbf{A}$ and vectors $\mathbf{x}, \mathbf{b}$ have a block structure:

$$\begin{bmatrix} \mathbf{A}_{zz} & \mathbf{A}_{z\theta} \\ \mathbf{A}_{\theta z} & \mathbf{A}_{\theta\theta} \end{bmatrix} \begin{bmatrix} \hat{\mathbf{U}}_z^* \\ \hat{\mathbf{U}}_\theta^* \end{bmatrix} = \begin{bmatrix} \mathbf{b}_z \\ \mathbf{b}_\theta \end{bmatrix}, \quad \text{where} \quad \begin{aligned} \mathbf{A}_{zz} &= (i f_h \alpha^2)\mathbf{I} - \mathbf{L_0} \\ \mathbf{A}_{z\theta} &= -\beta \mathbf{L_1} \\ &\ldots \end{aligned} \tag{SI48}$$

Code Implementation: Each block is constructed and then stacked together.

```
I = sp.eye(self.n, format="csr")
Azz = (1j * f_h * alpha**2) * I - self.L0; Azt = -beta * self.L1
Atz = -gamma * self.L0; Att = (1j * f_h * alpha**2) * I - delta * self.L1
...
A_top = sp.hstack([Azzb.tocsr(), Aztb.tocsr()])
A_bot = sp.hstack([Atzb.tocsr(), Attb.tocsr()])
A = sp.vstack([A_top, A_bot], format="csc")
```

Step 5: Imposing Boundary Conditions via Row Replacement

This is the most critical step for ensuring a physically correct solution. The matrix $\mathbf{A}$ as assembled above represents the ODEs everywhere, including at the boundaries. We must replace the ODE equations at the boundary nodes with new algebraic equations that directly enforce the physical boundary conditions.

Theory and Implementation: Consider the no-slip condition for axial velocity at the wall, $\hat{U}_z^*(1) = 0$. This corresponds to the last node, index `[-1]`. We want to enforce the simple algebraic equation $1 \cdot \hat{U}_{z,[-1]}^* = 0$. To do this, we modify the corresponding row in the matrix system (the `[-1]` row of the top block):

1. Zero out the entire row of the $\mathbf{A}_{zz}$ block.
2. Place a '1' on the diagonal of that row.
3. Zero out the corresponding row in the $\mathbf{A}_{z\theta}$ block.
4. Set the corresponding element of the right-hand-side vector $\mathbf{b}_z$ to zero.

This procedure effectively replaces the ODE at the wall with the desired boundary condition.

Code Implementation for No-Slip on U_z:

```
# For this row, the equation becomes 0*U_z[0] + ... + 1*U_z[-1] + 0*U_theta[...] = 0
Azzb[-1,:], Azzb[-1,-1], Aztb[-1,:], bz[-1] = 0.0, 1.0, 0.0, 0.0
```

A similar replacement is done for all four boundary conditions. The code uses the `.tolil()` sparse matrix format because it is efficient for changing the matrix structure, then converts back to `.tocsr()` for the solve step.

Step 6: Solving the Final Linear System

After embedding the boundary conditions, the result is a sparse, complex-valued, and non-singular linear system $\mathbf{Ax} = \mathbf{b}$.

Code Implementation: This system is solved using a highly optimized direct sparse solver from the SciPy library.

```
sol = spla.spsolve(A, b)
```

The resulting solution vector `sol` contains the concatenated velocity amplitudes, which are then separated.

```
return sol[:self.n], sol[self.n:] # Returns U_z_hat, U_theta_hat
```

This entire process is repeated for each harmonic of the input pressure waveform. The final time-domain velocity profiles are then synthesized by summing the contributions from each harmonic.

SI4 Detailed Grid-Independence Verification

To rigorously verify spectral convergence, we performed a successive-refinement study using Chebyshev–Gauss–Lobatto grids $N = \{60, 80, 100, 120, 140, 160, 180\}$.

Spectral discretization

For each N, the anisotropic Womersley system was solved in spectral collocation form. The multi-harmonic solution was reconstructed in time as

$$u_z(r,t) = \sum_{h=1}^{6} \hat{u}_{z,h}(r)\, e^{i2\pi ht}, \tag{SI49}$$

with harmonics identical to those used in the main main article. All parameters correspond to the thoracic aorta case ($R = 0.012\,\mathrm{m}$, $G_0 = 7000\,\mathrm{Pa/m}$, $\beta = 0.1$).

Reference solution and spectral-consistent evaluation

The solution computed at $N = 180$ was treated as the reference profile $u_z^{(180)}(r)$. To avoid interpolation artifacts, the reference solution was evaluated on each coarse grid using barycentric polynomial interpolation, which preserves spectral accuracy.

Residual definition

The pointwise residual was defined as

$$E_N(r_i) = \left| u_z^{(N)}(r_i) - u_z^{(180)}(r_i) \right|, \tag{SI50}$$

evaluated at the Chebyshev nodes of each grid. Residual profiles decrease uniformly with refinement. For $N \geq 140$, the residual magnitude is $\mathcal{O}(10^{-14})$ throughout the domain, approaching double-precision machine limits. No localized near-wall amplification is observed. These results confirm that $N = 150$ provides a spectrally converged solution for all quantities reported in the main article.
